# Line-of-Sight Probability Analysis of Underground Mining Visible Light Communication Diversity Schemes Under Random Receiver Orientation

**DOI:** 10.3390/s25092890

**Published:** 2025-05-03

**Authors:** Julián Solís, Iván Sánchez, Cesar Azurdia-Meza, Pablo Palacios Játiva, David Zabala-Blanco, Ali Dehghan Firoozabadi

**Affiliations:** 1 Department of Electrical Engineering, Universidad de Chile, Santiago 8370451, Chile; julian.solis@ug.uchile.cl (J.S.); cazurdia@ing.uchile.cl (C.A.-M.); 2 Department of Networking and Telecommunication Engineering, Universidad de las Américas, Quito 170503, Ecuador; 3 Escuela de Informática y Telecomunicaciones, Universidad Diego Portales, Santiago 8370190, Chile; pablo.palacios@mail.udp.cl; 4 Department of Computing and Industries, Universidad Católica del Maule, Talca 3466706, Chile; dzabala@ucm.cl; 5 Department of Electricity, Universidad Tecnológica Metropolitana, Av. José Pedro Alessandri 1242, Santiago 7800002, Chile; adehghanfirouzabadi@utem.cl

**Keywords:** line-of-sight (LoS) link, optical diversity schemes, optimal tilt angle, underground mining visible light communication (UM-VLC), visible light communication (VLC)

## Abstract

Visiblelightcommunication (VLC) is an emerging technology that offers an alternative to traditional wireless communications systems. However, the technology presents limitations related to the impact of the receiver’s orientation, which can significantly impact its performance. To address this issue, VLC systems use diversity schemes, such as transmitter and receiver diversity. In this paper, we derive an analytical expression for the probability of maintaining a line-of-sight (LoS) link in an underground mining visible light communication (UM-VLC) system with a receiver embedded in an object, such as a helmet, by considering user mobility. We show that the angle of incidence depends on the distance from the source and derive the probability accordingly for single-input single-output (SISO), multiple-input single-output (MISO), and single-input multiple-output cases (SIMO). Our results show that the analytical results fit with the simulated results. Furthermore, the resulting probabilities show that the angular position of the receiver significantly affects the channel’s quality, with the optimal position dependent on the field-of-view characteristics. These findings can provide an appropriate framework for receiver and transmitter diversity design through analytical expression.

## 1. Introduction

Visible light communication (VLC) is an emerging technology that poses an alternative to traditional physical communication technologies such as radio frequency (RF) communication. Due to its high frequency, it presents several advantages over RF communication, such as resistance to electromagnetic interference, an unlicensed spectrum, lower energy consumption, and reduced costs. The benefits come from the usage of energy-efficient light-emitting diodes (LEDs), which are now being deployed as the primary source of lighting.

A relevant use case for VLC is underground mining (UM) communications [1]. Visible light communication in underground mining (UM-VLC) is a promising solution due to its low cost and the latent necessity of lighting inside mines [2]. Although VLC solves many issues, such as interference and fading phenomena, highly directional propagation harms the system’s performance. The unpredictable receiver orientation and the user’s mobility frequently lead to a link misalignment. The misalignment of the link disrupts communication, causing link failures. To address these outages, different studies have studied the application of diversity techniques such as receiver diversity [3,4].

The existing proposed VLC implementations in mining environments require both mobility and a variable receiver orientation, which directly impact the availability of line-of-sight (LOS) links. Consequently, characterizing the availability of these links is a crucial element in understanding the system’s behavior. Although the impact of the receiver orientation has been studied extensively in the literature [5,6], research on random receiver orientations is limited by assumptions that do not accurately represent the underground mining scenario studied.

In the context of relevant and related work, the probability of a power outage has been studied under the case of a random transmitter orientation [7]. However, the receiver orientation has been considered constant and orthogonal to the ground plane. In [8,9], the power outage probability and the bit error rate for on–off keying (OOK) were studied under a uniform and Gaussian distribution of the receiving angle. However, the transmitter was considered orthogonal to the ground, and the distributions were not a function of the receiver’s position. In [10], the bit error rate and the signal-to-noise ratio distribution were considered for a case with arbitrary user positioning and an arbitrary orientation, and the optimum tilt angle was obtained. However, the article derived the effect of a random receiver orientation from fitting a known distribution. Likewise, [11] derived the bit error rate for DCO-OFDM with an arbitrary orientation by modeling the orientation and the channel using a Laplace distribution. However, the user positioning was maintained as fixed in the expression. In [12], the probability of a blockage and the probability of coverage were obtained by studying the shadowing generated by a cylinder. However, the transmitter was orthogonal to the ground, and the receiver was unaffected by a random orientation.

A random receiver orientation has also been studied in the context of non-orthogonal multiple-access (NOMA) schemes for VLC [13]. However, the reception angle was modeled using a normal distribution, which was also supposed to be independent of the radius. The bit error rate has been studied for single-input single-output (SISO) underground mining channels [14]. However, the receiving angle was considered to be independent of the user’s position. Finally, the power outage probability of a hybrid RF-VLC link was studied in a random receiver orientation [15]. However, the receiving angle was assumed to follow a normal distribution, similarly to in the NOMA study.

In this work, we investigate the effects of the receiver orientation from the perspective of an underground mining use case in SISO, multiple-input single-output (MISO), and single-input multiple-output (SIMO) scenarios. In particular, we investigate the case where the random receiver orientation’s density function is determined by its position with respect to the transmitter in a tunnel. The contributions of this paper can be summarized as follows:

- We develop an analytical framework to obtain the LoS probability of a mobile receiver with a random orientation given a rectangular cell for SISO, MISO, and SIMO cases.
- We obtain the closed analytical form of the LOS probability, given a uniform distribution of the receiver in the rectangular area and a uniform orientation of the user.
- We verify the analytical results through extensive simulation in all cases of interest.

The structure of this article is as follows. The MIMO-VLC model for use in underground environments and the impact of LoS communication links are presented in Section 2. The LoS probability and the angle of incidence are derived in Section 3. The analytical expressions of the SISO LoS probability, the SIMO LoS probability, and the MISO LoS probability are derived in Section 4, Section 5, and Section 6, respectively. The numerical and analytical results are presented in Section 7. Finally, conclusions are given in Section 8.

## 2. MIMO Channel Model

**Figure 1:**
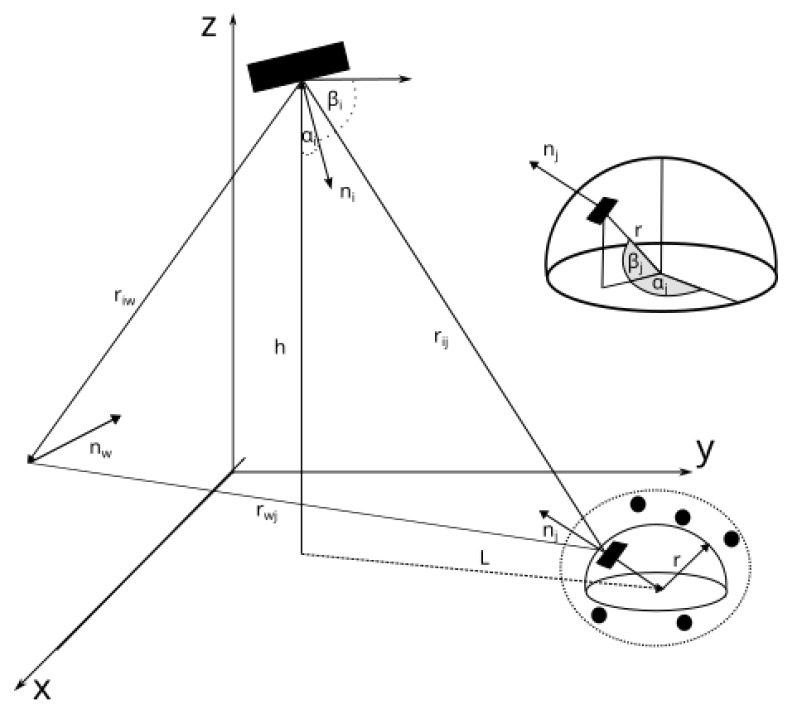
Geometrical representation of the evaluated scenario, where *L* represents the distance between the transmitter and the user in the XY plane and *h* represents the distance along the *z* axis.

The underground mining MIMO channel was modeled to account for multiple phenomena, such as reflections caused by irregular walls, scattering effects caused by dust particles, random shadowing caused by machinery entering the mine, and the random tilting of both the receiver and transmitter [16,17]. The tilt of the receiver and transmitter is defined by their corresponding azimuth angle α and elevation angle β. The geometry of the system is depicted in Figure 1. The orientation of the receiver and the transmitter in space is represented by their vectors:

(1) $$\begin{array}{cc} \; & n_{i}^{T}=\left[\cos\left(\alpha_{i}^{T}\right)\sin\left(\beta_{i}^{T}\right),\sin\left(\alpha_{i}^{T}\right)\sin\left(\beta_{i}^{T}\right),-\cos\left(\beta_{i}^{T}\right)\right],\\ \; & n_{j}^{R}=\left[\cos\left(\alpha_{j}^{R}\right)\sin\left(\beta_{j}^{R}\right),\sin\left(\alpha_{j}^{R}\right)\sin\left(\beta_{j}^{R}\right),\cos\left(\beta_{j}^{R}\right)\right], \end{array}$$

where $\alpha_{j}^{R}$ and $\beta_{j}^{R}$ correspond to the azimuthal and elevation angles of the receiver, and $\alpha_{i}^{T}$ and $\beta_{i}^{T}$ correspond to the azimuthal and elevation angles of the transmitter.

The channel model is characterized by a matrix of N×M, where *M* represents multiple transmitters and *N* represents multiple receivers. Each transmitter–receiver pair employs ray tracing to generate a channel that linearly sums the LoS component, the reflective components (NLoS), and the scattering components. The channel matrix is defined as follows:

(2) $$H=H_{LoS}+H_{sca}+H_{NLoS},$$

where $H_{LoS}$ is the LOS component, $H_{sca}$ is the scattering component, and $H_{NLoS}$ is the reflective component. $H_{LoS}$ is defined by the equation [2]

(3) $$H_{LoS}\left(i,j\right)=\frac{\left(m+1\right)A_{p}}{2\pi d_{ij}^{m+3}}{\left(r_{ij}\cdot n_{i}^{T}\right)}^{m}\left(r_{ij}\cdot n_{j}^{R}\right)G\left(\theta_{ij}\right)rect\left(\frac{\theta_{ij}}{\Theta }\right)P_{ij}H\left(\Delta_{ij}\right),$$

where $A_{p}$ represents the photodetector active area, *m* denotes the Lambertian order, $d_{ij}$ is the Euclidean distance between the receiver and the transmitter, Θ is the field of view, and $G(\theta_{ij})$ is the gain produced by the concentrator, defined by

(4) $$G\left(\theta_{ij}\right)=\left\{\begin{array}{c} \frac{\eta^{2}}{\sin^{2}\left(\Theta \right)},\;\mathrm{if}\;\theta_{ij}\leq \Theta \\ 0,\mathrm{else} \end{array}\right.,$$

where η is the gain and $\theta_{ij}$ is the angle between the receiver and the path between the receiver and the transmitter. $\Delta_{ij}$ is the dot product between $n_{i}^{T}$ and $r_{ij}$, and H(·) is the Heaviside function. Finally, $P_{ij}$ is the shadowing probability between the photodetector and the receiver. The shadowing probability is defined using a Poisson point process, where the expected probability of not encountering an obstacle during a period, t, is

(5) $$P_{ij}=e^{-\epsilon E(p_{v}^{ij})t},$$

with ϵ being the intensity parameter, *t* the expected time, and $E(p_{v})$ the expected probability of an obstacle causing shadowing [18].

(6) $$E\left(p_{v}^{ij}\right)=\int_{0}^{X}\int_{0}^{Y}\left({\;\int \int \;}_{w\geq 2d_{ij}\left(x_{v},y_{v}\right),h\geq s_{ij}\left(x_{v},y_{v}\right)}g\left(w,h\right)dwdh\right)f\left(x,y\right)dydx,$$

where *X* and *Y* are the horizontal and vertical dimensions of the tunnel, *w* is the width of the object, *h* is the height of the object, f(x,y) is the probability associated with the center of the object, and g(w,h) is the probability associated with the dimensions of the object. However, due to the cost of computing this equation multiple times over the simulation duration, a closed analytical form is obtained by approximating the probability density of the object dimensions using

(7) $$\begin{array}{cc} E(p_{v})= & E(E\left(p_{v}|w,h\right))\\ \; & =\sum_{w,h}\left(\int_{0}^{X}\int_{0}^{Y}1_{w\geq 2d\left(x_{v},y_{v}\right),h\geq s\left(x_{v},y_{v}\right)}f\left(x,y\right)dydx.\right)g\left(w,h\right). \end{array}$$

A closed analytical form of the probability can be found in Appendix A.

The non-line-of-sight (NLoS) component is the result of multiple reflections that can occur between the transmitter and the receiver. Hence, considering the NLoS component results in the recursive infinite summation of all the path combinations produced by the discrete reflectors. However, to simplify the calculation, assuming that the distance between the walls is considerable, we can limit the sum to one iteration. This relationship can be defined using the following equation:

(8) $$\begin{array}{cc} \; & H_{NLoS}\left(i,j\right)=\frac{\left(m+1\right)A_{p}}{2\pi }\sum_{w}\frac{A_{w}\rho_{w}}{d_{iw}^{m+1}d_{wj}^{3}}{\left(r_{iw}\cdot \hat{n_{i}}\right)}^{m}\cos\left(\theta_{iw}\right)\\ & \times \left(r_{wj}\cdot \hat{n_{w}}\right)\cos\left(\theta_{wj}\right)G\left(\theta_{wj}\right)rect\left(\frac{\theta_{wj}}{\Theta }\right), \end{array}$$

where $A_{w}$ corresponds to the area of the reflector and $\rho_{w}$ is the reflection coefficient of the wall. Similarly to the transmitter and receiver case, the reflector is characterized by a vector, $n_{w}^{W}$, which is associated with a random azimuth angle, $\alpha_{w}^{W}$, and an elevation, $\beta_{w}^{W}$, and the distance between the reflector and the transmitter or receiver is defined by $d_{iw}$ and $d_{wj}$, respectively. Finally, the scattering component is defined by

$$H_{sca}^{i,j}=\lim_{N\to \infty }\sum_{n=1}^{N}\frac{A_{p}\left(m+1\right)G_{n}\left(\mu \right)}{2\pi D_{i-n-j}^{2}}\cos^{m}\left(\phi_{i-S_{n}}\right)\cos\left(\theta_{S_{n}-j}\right)\times rect\left(\frac{\theta_{S_{n}-j}}{\Theta }\right),$$

where $D_{i-n-j}$ is the total distance covered by the rays from the transmitter to the receiver, $\phi_{i-S_{n}}$ is the angle between the scattering and the transmitter, $\theta_{S_{n}-j}$ is the angle between the scattering and the receiver, and $G_{n}\left(\mu \right)$ is the expected value of the scattering given the parameters

(9) $$\begin{array}{cc} \; & G_{n}\left(\mu \right)=\rho_{s}f_{sca}\left(\mu \right)/N. \end{array}$$

(10) $$\begin{array}{cc} \; & f_{sca}=\left(\frac{k_{m}}{k_{s}}p_{mie}\left(\mu \right)+\frac{k_{r}}{k_{s}}p_{ray}\left(\mu \right)\right)\sin\left(\mu \right). \end{array}$$

(11) $$\begin{array}{cc} \; & p_{ray}\left(\theta_{s}\right)=\frac{3[1+3\gamma +\left(1-\gamma \right)\cos^{2}\left(\theta_{s}\right)]}{16\pi (1+2\gamma )}. \end{array}$$

(12) $$\begin{array}{cc} \; & p_{mie}\left(\theta_{s}\right)=\frac{1-g^{2}}{4\pi }\left[\frac{1}{{\left(1+g^{2}-2g\cos\left(\theta_{s}\right)\right)}^{\frac{3}{2}}}+\frac{g(3\cos^{2}\theta_{s}-1)}{0.286{\left(1+g^{2}+0.286g\right)}^{\frac{5}{2}}}\right]. \end{array}$$

where γ, *g*, and *f* are atmospheric constants, $\rho_{s}$ is the reflection coefficient of the particles, μ is $\cos(\theta_{s})$, *N* is the number of particles, and $\theta_{s}$ is the angle of the scattering. The equations for $k_{r}$ and $k_{s}$ are [19]

(13) $$\begin{array}{cc} \; & k_{r}=\frac{24\pi^{3}}{\lambda^{4}N_{s}}\frac{{\left(n_{s}^{2}-1\right)}^{2}}{{\left(n_{s}^{2}+2\right)}^{2}}\frac{6+3\delta }{6-7\delta }\\ \; & k_{s}=N\frac{\lambda^{2}}{2\pi }\sum_{n=1}^{\infty }\left(2n+1\right)({\left|a_{n}\right|}^{2}+|b_{n}{|}^{2}),\\ \; & a_{n}=\frac{\Psi_{n}\left(x\right)\Psi_{n}\left(mx\right)-m\Psi_{n}{\left(x\right)}^{\prime }\Psi_{n}\left(mx\right)}{\xi_{n}\left(x\right)\xi_{n}^{\prime }\left(mx\right)-m\xi_{n}^{\prime }\left(x\right)\Psi_{n}\left(mx\right)},\\ \; & b_{n}=\frac{m\Psi_{n}\left(x\right)\Psi_{n}^{\prime }\left(mx\right)-\Psi_{n}^{\prime }\left(x\right)\Psi_{n}\left(mx\right)}{m\xi_{n}\left(x\right)\xi_{n}^{\prime }\left(mx\right)-\xi_{n}^{\prime }\left(x\right)\Psi_{n}\left(mx\right)}, \end{array}$$

where $x=\frac{2\pi \rho }{\lambda }$, with ρ being the radius of the particles, *m* the refractive coefficient, δ the depolarization factor of air, $N_{s}$ the molecular number density of air, and $n_{s}$ the refractive index of air. $\Psi_{n}$ and $\xi_{n}$ are the Bessel function and the first kind of Hankel function, respectively.

**Figure 2:**
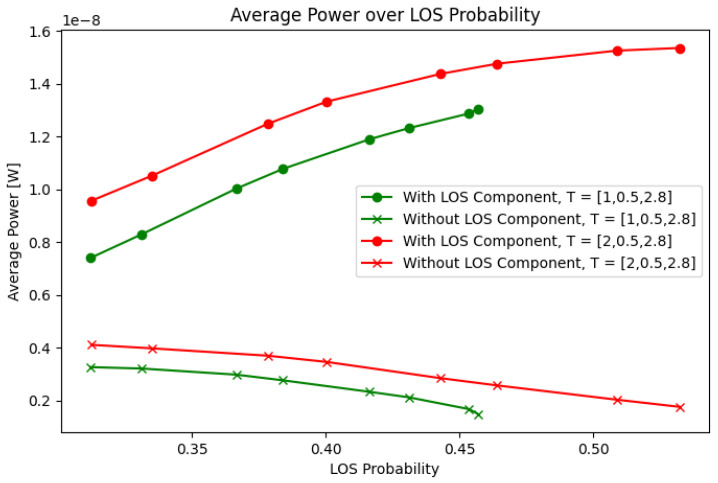
Average power from $\beta =20^{\circ }$ to $\beta =70^{\circ }$.

An important metric for assessing the effect of the receiving angle is the power outage probability. However, as will be shown in the following sections, the power outage probability cannot be obtained analytically, given the interdependence of the receiving angle with the mobility of the user and the orientation of the receiver. However, it is possible to obtain the LOS probability, which correlates strongly with the system’s average power. Given a variable angle of inclination for the receiver from $\beta_{r}=20^{\circ }$ to $\beta_{r}=70^{\circ }$, Figure 2 shows the average power compared to the LoS probability.

The average power and the LoS probability are obtained by simulating the channel at each point inside the tunnel by varying $\alpha_{j}^{R}$ between 0 and 2π. The systems and scenario parameters used for the simulation are presented in Table 1 and Table 2. The average power is obtained by computing the mean power of all the resulting DC channel values, and the LoS probability is obtained by taking the number of available LoS links and dividing them by the total number of links. The presented average power is further divided into a case with an LoS component and a case without an LoS component. The average for the case without an LoS component is computed by taking the mean of the DC power from all the corresponding phenomena within the channel bar the LoS component, even if an LoS component is available. Since we consider all the computed channel responses, the average power is equally conditioned by the distance between the receiver and the transmitter for all the varying inclination values.

The figure shows that the LoS probability and the average power in the tunnel are directly correlated when the LoS component is taken into account. Indeed, the correlation between the Los probability and the average power is r=0.9934. Based on this, we can conclude that obtaining a metric for the LoS probability given the present scenario would help predict the performance according to other metrics such as the power and optimize for the optimal angle.

## 3. Line-of-Sight Probability

The LoS probability is the probability that the receiver and the transmitter send data to each other using the LoS channel. To satisfy this condition, the gain from the concentrator must be greater than 0. The condition is satisfied as long as the reception angle is less than the total field of view. So, the probability is

(14) $$P\left(\mathrm{Line}-\mathrm{of}-\mathrm{Sight}\;\mathrm{Probability}\right)=P\left(\theta_{ij}\leq \Theta \right),$$

where $\theta_{ij}$ can be defined by the inverse cosine:

(15) $$\theta_{ij}=\cos^{-1}\left(\frac{n_{ij}\cdot n_{j}^{R}}{\left|\right|n_{ij}{\left|\right|}^{2}}\right),$$

and ||·|| is the Euclidean norm, and $n_{ij}$ is the vector from the transmitter to the receiver, which is

(16) $$n_{ij}=\left[\begin{array}{c} L\cos\left(\theta \right)+r\cos\left(\alpha_{j}^{R}\right)\sin\left(\beta_{j}^{R}\right)\;\\ -L\sin\left(\theta \right)+r\sin\left(\alpha_{j}^{R}\right)\sin\left(\beta_{j}^{R}\right)\;\\ h+r\cos(\beta_{j}^{R}) \end{array}\right].$$

where *r* is the radius of the sphere around the receiver that parameterizes the position of the receiver, *L* is the distance from the transmitter to the receiver in the X–Y plane, and *h* is the height difference between the transmitter and the receiver.

Unlike in the orthogonal case ($\beta_{j}^{R}=0^{\circ }$), the tilt produced by α and β involves a non-circular area of possible transmitter positions, which depends on the angle α. Since the azimuth angle changes with the rotations of the receiver, the probability of reception given a certain coordinate of the center of the receiver is a function of the radius *L*. The density probability function of the receiving angle is as follows:

(17) $$f_{\theta }\left(L\right)=\frac{1}{2\pi }\frac{\sin\left(\theta \right)\sqrt{L^{2}+h^{2}+r^{2}+2hr\cos\left(\beta \right)}}{\sqrt{L^{2}\sin^{2}\left(\beta \right)-\left(\sqrt{L^{2}+h^{2}+r^{2}+2hr\cos(\beta }\right)}{\cos\left(\theta \right)-h\cos\left(\beta \right)-r)}^{2}}.$$

**Figure 3:**
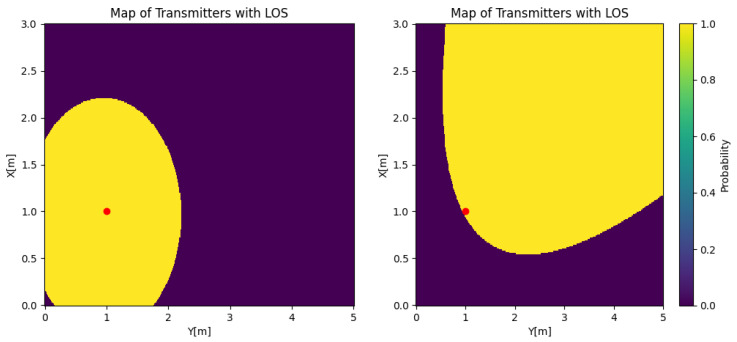
Orthogonal case for $\Theta =45^{\circ }$ with $\beta =0^{\circ }$ on the left and $\beta =45^{\circ }$ on the right.

As using the probability density function has an important difference to the orthogonal case, the analysis of the system changes. Since *L* is not deterministic, the value of the function changes with respect to the probability given by the mobility of the user, and the power will depend on both α and *L*. To illustrate the difference between both systems, Figure 3 shows the set of transmitters with their LoS probability for the orthogonal case (β=0) and the non-orthogonal case ($\beta =45^{\circ }$, $\alpha =45^{\circ }$). The receiver is positioned at [1,1,1.8], which is represented by the red dot in both figures. The geometry of both cases shows the reason behind the complexity of obtaining the LoS probability. In the orthogonal case, the probability can be calculated by taking the indicator function over the area of potential transmitters. However, in the studied case, each coordinate will have an LoS probability that can be understood as the integration over the realization of rotating the receiver around itself. In that case, points closer to the origin will still have a probability of one, as they will always have the link in each rotation, but points far from the receiver will be visited with less frequency.

## 4. SISO LoS Probability

The LoS probability can be obtained using Equation (14) by combining $\theta_{ij}$ and applying cosine to both sides. Since $\theta_{ij}$ belongs to the interval [0,π], the cosine is monotonically decreasing. The consistent decline enables us to express Equation (14) by utilizing

(18) $$\begin{array}{cc} P(\mathrm{In}\;\mathrm{FOV}) & =P\left(\theta_{ij}\leq \Theta \right)+P\left(2\pi -\theta_{ij}\leq 2\pi -\Theta \right)\\ \; & =2P(\cos\left(\theta_{ij}\right)\geq \cos\left(\Theta \right))\\ \; & =2P(\frac{n_{ij}n_{j}^{R}}{\left|\right|n_{ij}{\left|\right|}^{2}}\geq \cos\left(\Theta \right)), \end{array}$$

where $\cos\left(\theta_{ij}\right)=\cos\left(2\pi -\theta_{ij}\right)$ due to the parity of the cosine function.

The left argument of the probability is obtained using the definitions of $n_{ij}$ and $n_{j}^{R}$. The resulting expressions for the norm and the dot product are

(19) $$\begin{array}{cc} \left|\right|n_{ij}{\left|\right|}^{2} & =\sqrt{L^{2}+r^{2}+h^{2}+2hr\cos\left(\beta \right)+2rL\left(\cos\left(\theta \right)\cos\left(\alpha \right)\sin\left(\beta \right)-\sin\left(\theta \right)\sin\left(\alpha \right)\sin\left(\beta \right)\right)}\\ \; & \approx \sqrt{L^{2}+r^{2}+h^{2}+2hr\cos\left(\beta \right)},\\ n_{ij}\hat{n_{j}} & =L\cos(\theta )\cos(\alpha )\sin(\beta )-L\sin(\theta )\sin(\alpha )\sin(\beta )+r+h\cos(\beta )\\ \; & =L\sin(\beta )\cos(\alpha +\theta )+r+h\cos(\beta ), \end{array}$$

where the approximation of the norm holds as long as *r* is significantly smaller than both *h* and *L*. Using both expressions, we can rewrite the probability as

(20) $$\begin{array}{cc} \; & 2P\left(\theta_{ij}\leq \Theta \right)=2P\left(\cos\left(\alpha +\theta \right)\geq \frac{\cos\left(\Theta \right)\sqrt{L^{2}+r^{2}+h^{2}+2hr\cos\left(\beta \right)}-r-h\cos\left(\beta \right)}{L\sin(\beta )}\right),\\ \; & P\left(\theta_{ij}\leq \Theta \right)=P\left(\alpha \leq \cos^{-1}\left(\frac{\cos\left(\Theta \right)\sqrt{L^{2}+r^{2}+h^{2}+2hr\cos\left(\beta \right)}-r-h\cos\left(\beta \right)}{L\sin(\beta )}\right)-\theta \right). \end{array}$$

The above equation has three random variables, which dictate the position of the receiver, determined by θ and *L*, and the orientation of the receiver, which is determined by α. Since the receiver’s orientation is assumed to be independent of its position, we can obtain the expected LOS probability using the following equation:

(21) $$\begin{array}{cc} \; & E_{L,\theta }\left(P\left(\mathrm{FOV}|L,\theta \right)\right)=\int_{L,\theta }\left(2P\left(\theta_{ij}\leq \Theta |L,\theta \right)f_{L,\theta }\right)dLd\theta ,\\ \; & P\left(\theta_{ij}\leq \Theta |L,\theta \right)=\int_{0}^{\cos^{-1}\left(\frac{\cos\left(\Theta \right)\sqrt{L^{2}+r^{2}+h^{2}+2hr\cos\left(\beta \right)}-r-h\cos\left(\beta \right)}{L\sin(\beta )}\right)}f_{\alpha }\left(\alpha \right)d\alpha , \end{array}$$

where $f_{\alpha }$ is the probability density function of the receiver’s orientation, assumed to be uniform within the interval [0,2π], and $f_{L,\theta }$ is the probability density function of the receiver’s position, assumed to be uniform too. Due to the use of the radius and the angle, and given the rectangular boundaries of the system, the spatial density function has to be rewritten to

(22) $$\begin{array}{c} f_{r,\theta }^{2n+1}\left(r,\theta \right)=\left\{\begin{array}{c} \frac{2r}{{\left(\Delta D_{n//2}\right)}^{2}\tan\left(\theta_{cr}^{n}\right)}1_{r\leq \frac{\Delta X_{n//2}}{\cos(\theta^{\prime })}}P\left(S_{2n+1}\right),\;\mathrm{if}\;n\;\left(\mathrm{mod}\;2\right)=0\\ \frac{2r}{{\left(\Delta Y_{n//2}\right)}^{2}\tan\left(\theta_{cr}^{n}\right)}1_{r\leq \frac{\Delta Y_{n//2}}{\cos(\theta^{\prime })}}P\left(S_{2n+1}\right),\;\mathrm{if}\;n\;\left(\mathrm{mod}\;2\right)=1 \end{array}\right., \end{array}$$

(23) $$\begin{array}{c} f_{r,\theta }^{2n}\left(r,\theta \right)=\left\{\begin{array}{c} \frac{2r}{{\left(\Delta Y_{n//2}\right)}^{2}\cot\left(\theta_{cr}^{n}\right)}1_{r\leq \frac{\Delta Y_{n//2}}{\sin(\theta^{\prime })}}P\left(S_{2n}\right),\;\mathrm{if}\;n\;\left(\mathrm{mod}\;2\right)=0\\ \frac{2r}{{\left(\Delta D_{n//2}\right)}^{2}\cot\left(\theta_{cr}^{n}\right)}1_{r\leq \frac{\Delta X_{n//2}}{\sin(\theta^{\prime })}}P\left(S_{2n}\right),\;\mathrm{if}\;n\;\left(\mathrm{mod}\;2\right)=1 \end{array}\right., \end{array}$$

where *n* is an index from 1 to 8 that represents the different triangular partitions of the corresponding rectangle. The resulting integral is the equation

(24) $$\begin{array}{cc} \; & \int_{\alpha }p\left(\theta_{ij}\leq \Theta |L,\theta \right)=\int_{0}^{\cos^{-1}\left(\frac{\cos\left(\Theta \right)\sqrt{L^{2}+b^{2}}-a}{L\sin(\beta )}\right)}2\frac{1}{2\pi }d\alpha \\ \; & =\left\{\begin{array}{c} 0,\;\mathrm{if}\;\frac{\cos\left(\Theta \right)\sqrt{L^{2}+b^{2}}-a}{L\sin(\beta )}\geq 1\\ 1,\;\mathrm{if}\;\frac{\cos\left(\Theta \right)\sqrt{L^{2}+b^{2}}-a}{L\sin(\beta )}\leq -1\\ \frac{1}{\pi }\cos^{-1}\left(\frac{\cos\left(\Theta \right)\sqrt{L^{2}+b^{2}}-a}{L\sin(\beta )}\right),\;\mathrm{else}, \end{array}\right. \end{array}$$

where $b=\sqrt{r^{2}+h^{2}+2hr\cos\left(\beta \right)}$ and a=r+hcos(β).

The inverse cosine can be approximated by using an odd number of linear approximations that are symmetric to the center. Each linear approximation will be associated with a slope, $a_{k}$, and an offset, $b_{k}$. Linear approximations represent an interval of the function. These intervals can be found by solving the following expression:

(25) $$\frac{\cos\left(\Theta \right)\sqrt{L^{2}+b^{2}}-a}{L\sin(\beta )}≷k_{j}.$$

The orientation of the inequality depends on the sign of $\cos\left(\Theta \right)\sqrt{L^{2}+b^{2}}-a$. If the sign is positive, then the thresholds $k_{j}$ represent either the lower bound from $\left[-\infty ,L_{o}\right]$ or the upper bounds from $\left[L_{o},\infty \right]$, where $L_{o}$ represents the minimum of the function if it exists. If the sign is negative, then the thresholds represent the upper bound. Because of its quadratic form, the equation can have zero to two feasible solutions over zero that follow the following solution:

(26) $$L_{k_{j}}=\left\{\begin{array}{c} \frac{ka\sin\left(\beta \right)\pm \sqrt{k^{2}a^{2}\sin^{2}\left(\beta \right)-\left(\cos^{2}\left(\Theta \right)-k\sin^{2}\left(\beta \right)\right)\left(b^{2}\cos^{2}\left(\Theta \right)-a^{2}\right)}}{\cos^{2}\left(\Theta \right)-k\sin^{2}\left(\beta \right)},\cos^{2}\left(\Theta \right)-k\sin^{2}\left(\beta \right)\neq 0\\ \frac{-(\cos^{2}\left(\Theta \right)b^{2}-a^{2}}{2ka\sin(\beta ))},\cos^{2}\left(\Theta \right)-k\sin^{2}\left(\beta \right)=0. \end{array}\right.$$

The intervals are generated depending on the number of solutions. If $L_{k_{j}}$ has zero solutions and $L_{k_{j-1}}$ has a solution, then $k_{j}$ is valid in the interval $\left[L_{k_{j-1}},+\infty \right]$. If $L_{k_{j-1}}$ has zero solutions, then $k_{j}$ is skipped. If $L_{k_{j}}$ has one solution, then $k_{j}$ is valid in the interval $\left[L_{k_{j-1}},L_{k_{j}}\right]$. If it has two positive solutions, then $k_{j}$ is valid in the interval $\left[L_{k_{j}}^{1},L_{k_{j-1}}^{1}\right]$ and the interval $\left[L_{k_{j-1}}^{2},L_{k_{j}}^{2}\right]$.

Using the solutions, we can form a succession of solutions such that the nth element of the solution is associated with some $k_{n}$ constant that corresponds to the approximation from $L_{n}$ to $L_{n+1}$, the values of which depend on the solutions for the previous equation. Using this sequence, we can rewrite the integral as the sum of the intervals that are associated with each $k_{n}$ over the sum of the different triangles that compose the probability distribution. The resulting equation is

(27) $$\begin{array}{cc} \; & E\left(P\left(\theta_{ij}\leq \Theta \right)\right)=\sum_{m=1}^{M}\sum_{n=1}^{4}\int_{\left(n-1\right)\frac{\pi }{2}}^{\theta_{cr}^{n}}\int_{L_{m-1}}^{L_{m}}g_{m}\left(L,\theta \right)f_{L,\theta }^{2n+1}dLd\theta +\int_{\theta_{cr}^{n}}^{n\frac{\pi }{2}}\int_{L_{m-1}}^{L_{m}}g_{m}\left(L,\theta \right)f_{L,\theta }^{2n}dLd\theta .\\ \; & g_{m}\left(L,\theta \right)=\frac{a_{m}}{\pi }\cos^{-1}\left(\frac{\cos\left(\Theta \right)\sqrt{L^{2}+b^{2}}-a}{L\sin(\beta )}\right)+\frac{b_{m}}{\pi }\\ \; & g_{1}\left(L,\theta \right)=1_{\cos\left(\Theta \right)\sqrt{L^{2}+b^{2}}-a<0} \end{array}$$

The integral can be divided into the 2n and 2n+1 cases, assuming that $g_{1}\left(L,\theta \right)$ is equivalent to having $a_{1}=0$ and $b_{1}=1_{\cos\left(\Theta \right)\sqrt{L^{2}+b^{2}}-a<0}$. Furthermore, the 2n case corresponds to the density functions of the tangent denominator, while the 2n+1 case corresponds to the density functions of the cotangent denominator. For the 2n+1 case, the integral is

(28) $$\begin{array}{cc} \; & I_{2n+1,m}=\int_{L,\theta }\cos^{-1}\left(\frac{\cos\left(\Theta \right)\sqrt{L^{2}+b^{2}}-a}{L\sin(\beta )}\right)f_{L,\theta }^{2n+1}dLd\theta =\\ \; & \int_{f_{2n+1}\left(\left(n-1\right)\frac{\pi }{2},L_{m-1},\theta_{cr}^{n}\right)}^{\theta_{cr}^{n}}\int_{L_{m-1}}^{L_{m}}\left[a_{m}\left(\frac{\cos\left(\Theta \right)\sqrt{L^{2}+b^{2}}-a}{L\sin(\beta )}\right)+b_{m}\right]\frac{2L}{{\left(\Delta X_{n}\right)}^{2}\tan\left(\theta_{cr}^{n}\right)}\\ \; & 1_{r\leq \frac{\Delta X_{n}}{\cos(\theta^{\prime })}}P\left(S_{2n+1}\right)dLd\theta . \end{array}$$

with $\Delta X_{n}=\Delta D_{n//2}1_{mod(n,2)=0}+\Delta Y_{n//2}1_{mod(n,2)=1}$. The function $f(\left(n-1\right)\frac{\pi }{2},L_{m-1})$ obeys

(29) $$f_{2n+1}\left(\left(n-1\right)\frac{\pi }{2},L_{m-1},\theta_{cr}^{n}\right)=\min\left(\tan^{-1}\left(\frac{\Delta X_{n}}{L_{m-1}}\right)+\left(n-1\right)\frac{\pi }{2},\theta_{cr}^{n}\right),$$

For the purpose of notation, it will be called $\theta_{n}^{L_{m-1}}$.

The integral solution consists of the following equations:

(30) $$\begin{array}{cc} \; & I_{2n+1,m}=I_{2n+1,m,1}1_{L_{m}<\Delta X_{n}}+I_{2n+1,m,2}1_{L_{m}>\frac{\Delta X_{n}}{\cos(\theta_{cr}^{n})}}+I_{2n+1,m,3}1_{L_{m}>\Delta X_{n}}1_{L_{m}<\frac{\Delta X_{n}}{\cos(\theta_{cr}^{n})}},\\ \; & I_{2n+1,m,3}=I_{2n+1,m,1}\left(\bar{\theta },\theta_{cr}^{n}\right)+I_{2n+1,m,2}\left(\theta_{n}^{L_{m-1}},\bar{\theta }\right)\\ \; & \bar{\theta }=\cos^{-1}\left(\frac{\Delta X_{n}}{L_{m}}\right), \end{array}$$

where $I_{2n+1,m,1}$ integrates an interval completely inside the rectangular boundary, $I_{2n+1,m,2}$ integrates an interval where the upper bound is completely outside of the rectangular boundary for every angle, and $I_{2n+1,m,3}$ integrates an interval where $\bar{\theta }$ defines whether the upper boundary is inside or outside of the boundary. The subterm $I_{2n+1,m,1}$ is

(31) $$\begin{array}{cc} \; & I_{2n+1,m,1}=\frac{2P(S_{2n+1})}{{\left(\Delta X_{n}\right)}^{2}\tan\left(\theta_{cr}^{n}\right)}\left(\theta_{cr}^{n}-\theta_{n}^{L_{m-1}}\right)\left(\Lambda_{m}\left(L_{m}\right)-\Lambda_{m}\left(L_{m-1}\right)\right)\\ \; & \Lambda_{m}\left(L\right)=a_{k}(\frac{\cos(\Theta )}{\sin(\beta )}\frac{1}{2}\left(L_{m}\sqrt{L_{m}^{2}+b^{2}}+b^{2}ln\left(\sqrt{L_{m}^{2}+b^{2}}+L_{m}\right)-\frac{a}{\sin(\beta )}L_{m}\right)+\\ \; & \frac{b_{m}}{2}L_{m}^{2}. \end{array}$$

The subterm $I_{2n+1,m,2}$, with $\sigma_{n}=\frac{\Delta X_{n}}{b}$, is given in Appendix B.

The integral of the even case follows the following equations:

(32) $$\begin{array}{cc} \; & I_{2n,m}=I_{2n,m,1}1_{L_{m}<\Delta X_{n}}+I_{2n,m,2}1_{L_{m}>\frac{\Delta X_{n}}{\sin(\theta_{cr}^{n})}}+I_{2n,m,3}1_{L_{m}>\Delta X_{n}}1_{L_{m}<\frac{\Delta X_{n}}{\sin(\theta_{cr}^{n})}},\\ \; & I_{2n+1,m,3}=I_{2n,m,2}\left(\bar{\theta },\theta_{cr}^{n}\right)+I_{2n,m,1}\left(\theta_{n}^{L_{m-1}},\bar{\theta }\right)\\ \; & \bar{\theta }=\sin^{-1}\left(\frac{\Delta X_{n}}{L_{m}}\right), \end{array}$$

where the subterm $I_{2n+1,m,1}$ is

(33) $$\begin{array}{cc} \; & I_{2n,m,1}=\frac{2P(S_{2n+1})}{{\left(\Delta X_{n}\right)}^{2}\cot\left(\theta_{cr}^{n}\right)}\left(\theta_{cr}^{n}-\theta_{n}^{L_{m-1}}\right)\left(\Lambda_{m}\left(L_{m}\right)-\Lambda_{m}\left(L_{m-1}\right)\right)\\ \; & \Lambda_{m}\left(L\right)=a_{k}(\frac{\cos(\Theta )}{\sin(\beta )}\frac{1}{2}\left(L_{m}\sqrt{L_{m}^{2}+b^{2}}+b^{2}ln\left(\sqrt{L_{m}^{2}+b^{2}}+L_{m}\right)-\frac{a}{\sin(\beta )}L_{m}\right)+\\ \; & \frac{b_{m}}{2}L_{m}^{2}, \end{array}$$

and the subterm $I_{2n,m,2}$ is given in Appendix B.

## 5. SIMO LoS Probability

The single-input multiple-output case includes $N_{r}$ receivers with an angular offset, $\alpha_{j}$, such as $\alpha_{0}=0$. Let us call $\alpha_{j}^{\prime }$ the sum of the probabilistic α and the offset. The dot product between $n_{ij}$ and $n_{j}$ will be

(34) $$\begin{array}{cc} \; & n_{ij}\hat{n_{j}}=L\cos\left(\theta \right)\cos\left(\alpha^{\prime }\right)\sin\left(\beta \right)-L\sin\left(\theta \right)\sin\left(\alpha^{\prime }\right)\sin\left(\beta \right)+r+h\cos\left(\beta \right)\\ \; & =L\sin\left(\beta \right)\cos\left(\alpha^{\prime }+\theta \right)+r+h\cos\left(\beta \right).\\ \; & \alpha^{\prime }=\alpha +\bar{\alpha } \end{array}$$

Obtaining the probability of $n_{t}$ receivers communicating during the same period involves intersecting the intervals produced by each $\alpha_{j}^{\prime }$. The intervals produced by each $\alpha_{j}^{\prime }$ are in the form of

(35) $$\alpha \in [-\cos^{-1}\left(u_{0}\left(L,\theta \right)\right)-\alpha_{j},\cos^{-1}\left(u_{0}\left(L,\theta \right)\right)-\alpha_{j}],$$

where $u_{0}\left(L,\theta \right)$ is the argument of the inverse, or

(36) $$u_{0}\left(L,\theta \right)=\frac{\cos\left(\Theta \right)\sqrt{L^{2}+r^{2}+h^{2}+2hr\cos\left(\beta \right)}-r-h\cos\left(\beta \right)}{L\sin(\beta )}.$$

Since α∈[−π,π] and the arguments of the intervals mentioned previously can exist outside of the interval, to solve this, we introduce the following transformation:

(37) $$\begin{array}{cc} \; & \alpha_{j}^{-}=\left(-\cos^{-1}\left(u_{0}\left(L,\theta \right)\right)-\alpha_{j}+\pi \right)\;\left(\mathrm{mod}\;2\pi \right)-\pi ,\\ \; & \alpha_{j}^{+}=\cos^{-1}\left(u_{0}\left(L,\theta \right)\right)-\alpha_{j}+\pi \;\left(\mathrm{mod}\;2\pi \right)-\pi . \end{array}$$

To obtain the points where the modulus cycles back to 0 or 2π, we need to find the solutions to the equation

(38) $$u_{0}\left(L_{j},\theta \right)=\cos\left(\alpha_{j}\right),$$

with $L_{j}$ being the solution to the equation. The equation has zero to two solutions. The possible cases are the following:

- If the equation has zero solutions, then the argument of the modulus either belongs to [−π,π] or is always below the interval. If $\alpha_{j}\leq \pi$, then it is the former; if $\alpha_{j}\geq \pi$, then it is the latter.
- If the equation has one solution, then it is either from the upper bound or the lower bound. If $\alpha_{j}\leq \pi$, then it is the former; if $\alpha_{j}\geq \pi$, then it is the latter.
- If the equation has two solutions, then one of the bounds oscillates around −π. If $\alpha_{j}\leq \pi$, then it goes below π from $L_{j}^{1}$ to $L_{j}^{2}$; if $\alpha_{j}\geq \pi$, then it goes above π from $L_{j}^{1}$ to $L_{j}^{2}$.

Without the loss of generality, the solution is always a pair, $L_{j}^{1}$ and $L_{j}^{2}$, where $L_{j}^{1}\leq L_{j}^{2}$. If there are one or fewer solutions, then $L_{j}^{1}$ goes to −∞. If there are zero solutions, then $L_{j}^{2}$ goes to −∞ too. Then, the intervals can be generalized using

(39) $$\begin{array}{ccc} \; & \alpha \in [-\cos^{-1}\left(u_{0}\left(L,\theta \right)\right)-\alpha_{j},\cos^{-1}\left(u_{0}\left(L,\theta \right)\right)-\alpha_{j}], & \alpha_{j}\leq \pi ,L\leq L_{j}^{1}\\ \; & \alpha \in \left[-\pi ,\cos^{-1}\left(u_{0}\left(L,\theta \right)\right)-\alpha_{j}\right]\cup \left[-\cos^{-1}\left(u_{0}\left(L,\theta \right)\right)-\alpha_{j}^{2\pi },\pi \right], & \alpha_{j}\leq \pi ,L_{j}^{1}\leq L\leq L_{j}^{2}\\ \; & \alpha \in [-\cos^{-1}\left(u_{0}\left(L,\theta \right)\right)-\alpha_{j},\cos^{-1}\left(u_{0}\left(L,\theta \right)\right)-\alpha_{j}], & \alpha_{j}\leq \pi ,L_{j}^{2}\leq L\\ \; & \alpha \in [\cos^{-1}\left(u_{0}\left(L,\theta \right)\right)-\alpha_{j}^{2\pi },-\cos^{-1}\left(u_{0}\left(L,\theta \right)\right)-\alpha_{j}^{2\pi }], & \alpha_{j}\geq \pi ,L\leq L_{j}^{1}\\ \; & \alpha \in \left[-\pi ,\cos^{-1}\left(u_{0}\left(L,\theta \right)\right)-\alpha_{j}\right]\cup \left[-\cos^{-1}\left(u_{0}\left(L,\theta \right)\right)-\alpha_{j}^{2\pi },\pi \right], & \alpha_{j}\geq \pi ,L_{j}^{1}\leq L\leq L_{j}^{2}\\ \; & \alpha \in [-\cos^{-1}\left(u_{0}\left(L,\theta \right)\right)-\alpha_{j}^{2\pi },\cos^{-1}\left(u_{0}\left(L,\theta \right)\right)-\alpha_{j}^{2\pi }], & \alpha_{j}\geq \pi ,L_{j}^{2}\leq L \end{array}$$

where $\alpha_{j}^{2\pi }=2\pi -\alpha_{j}$.

Similarly to in the SISO case, we can form a succession of distances to the center such that the nth element of the sequence corresponds to an interval, $L_{n-1}$ to $L_{n}$, such that both $L_{n-1}$ and $L_{n}$ are solutions for some pair of receivers. Let us assume that the first *M* receivers have non-divergent interval solutions, where $M_{0}$ corresponds to those which are not subject to a modulus and $M_{1}$ to those which are. Finally, the last *N* receivers have divergent interval solutions, which means that their lower bounds have a modulus applied to them. If N>0, then the resulting intervals are

(40) $$\begin{array}{cc} \; & \alpha \in [-\cos^{-1}\left(u_{0}\left(L,\theta \right)\right)-\alpha_{n,1},\cos^{-1}\left(u_{0}\left(L,\theta \right)\right)-\alpha_{n,2}]\\ \; & \cup [-\cos^{-1}\left(u_{0}\left(L,\theta \right)\right)-\alpha_{n,3},\cos^{-1}\left(u_{0}\left(L,\theta \right)\right)-\alpha_{n,4}]\\ \; & \alpha_{n,1}=\min\left(\alpha_{0},..\alpha_{M_{0}},\alpha_{M_{0}+1}-2\pi ,...,\alpha_{M_{1}}-2\pi \right)\\ \; & \alpha_{n,2}=\max\left(\alpha_{0},...,\alpha_{M+N}-2\pi \right)\\ \; & \alpha_{n,3}=\min\left(\alpha_{0},...,\alpha_{M+N}-2\pi \right)\\ \; & \alpha_{n,4}=\max\left(\alpha_{0},..\alpha_{M_{0}},\alpha_{M_{0}+1}-2\pi ,...,\alpha_{M_{1}}-2\pi \right) \end{array}$$

Since $\alpha_{0}$ is 0, M≥1. For the union to be between two non-intersecting intervals, the condition $\cos^{-1}\left(u_{0}\left(L,\theta \right)\right)\leq \frac{\alpha_{n,3}-\alpha_{n,2}}{2}$ must be satisfied, which is always true given the fact that the α associated with the minimum in $\alpha_{n,3}$ is always bigger than α associated with the maximum in $\alpha_{n,2}$. If N=0, then

(41) $$\begin{array}{cc} \; & \alpha \in [-\cos^{-1}\left(u_{0}\left(L,\theta \right)\right)-\alpha_{n,1},\cos^{-1}\left(u_{0}\left(L,\theta \right)\right)-\alpha_{n,2}]\\ \; & \alpha_{n,1}=\min\left(\alpha_{0},..\alpha_{M_{0}},\alpha_{M_{0}+1}-2\pi ,...,\alpha_{M_{1}}-2\pi \right)\\ \; & \alpha_{n,2}=\max\left(\alpha_{0},..\alpha_{M_{0}},\alpha_{M_{0}+1}-2\pi ,...,\alpha_{M_{1}}-2\pi \right) \end{array}$$

Using the succession of intervals χ, we can compute the integral by using the sum

(42) $$\begin{array}{cc} \; & E(P\left(\theta_{i1}\leq \Theta ,...,\theta_{i,N_{t}}\leq \Theta \right))=\\ \; & \sum_{n=1}^{\left|\chi \right|}\sum_{m=1}^{M}\sum_{l=1}^{4}\int_{(l-1)\pi }^{\theta_{cr}^{l}}\int_{L_{m-1}}^{L_{m}}[\left(g_{m}\left(L,\theta \right)+\frac{\alpha_{n,2}-\alpha_{n,1}}{\pi }\right)1_{\cos^{-1}\left(u_{0}\left(L,\theta \right)\right)\geq 0.5\left(\alpha_{n,2}-\alpha_{n,1}\right))}\\ \; & +\left(g_{m}\left(L,\theta \right)+\frac{\alpha_{n,4}-\alpha_{n,3}}{\pi }\right)1_{\cos^{-1}(u_{0}\left(L,\theta \right)\geq 0.5\left(\alpha_{n,4}-\alpha_{n,3}\right)}1_{N_{br}>0}]1_{L<L_{n},L>L_{n-1}}f_{L,\theta }^{2l+1}dLd\theta +\\ \; & \int_{\theta_{cr}^{l}}^{l\pi }\int_{L_{m-1}}^{L_{m}}[\left(g_{m}\left(L,\theta \right)+\frac{\alpha_{n,2}-\alpha_{n,1}}{\pi }\right)1_{\cos^{-1}\left(u_{0}\left(L,\theta \right)\right)\geq 0.5\left(\alpha_{n,2}-\alpha_{n,1}\right))}\\ \; & \left(g_{m}\left(L,\theta \right)+\frac{\alpha_{n,4}-\alpha_{n,3}}{\pi }\right)1_{\cos^{-1}(u_{0}\left(L,\theta \right)\geq 0.5\left(\alpha_{n,4}-\alpha_{n,3}\right)}1_{N_{br}>0}]1_{L<L_{n},L>L_{n-1}}f_{L,\theta }^{2l}dLd\theta . \end{array}$$

with $N_{br}$ being the number of receivers that have disjoint intervals of α. The inequality $\cos^{-1}\left(u_{0}\left(L,\theta \right)\right)\geq 0.5\left(\alpha_{n,k}-\alpha_{n,k-1}\right))$ has zero to two solutions and can be trivially solved within each interval, *n*. The solution to the integral of each $\alpha_{n,k}$ is equivalent to the integral of $g_{1}\left(L,\theta \right)$ multiplied by $\alpha_{n,k}$.

## 6. MISO LOS Probability

For the MISO case, we assume that the $n^{th}$ transmitter is at a distance, $d_{i}$, from the first transmitter, with an angle of $\phi_{i}$. The general formula for the dot product between $n_{ij}$ and $n_{j}$ is

(43) $$\begin{array}{cc} \; & n_{ij}\hat{n_{j}}=\left(L+d\cos\left(\phi_{i}\right)\right)\cos\left(\theta \right)\cos\left(\alpha \right)\sin\left(\beta \right)+\left(L+d\sin\left(\phi_{i}\right)\right)\sin\left(\theta \right)\sin\left(\alpha \right)\sin\left(\beta \right)+r+h\cos\left(\beta \right) \end{array}$$

To simplify the expression, we use $\phi_{i}=0$. The case with $\phi_{i}\neq 0$ has a similar solution but was not evaluated. If $\phi_{i}=0$, then the norm and the dot product are

(44) $$\begin{array}{cc} \; & n_{ij}\hat{n_{j}}=\sqrt{L^{2}+d^{2}+2dL\cos\left(\theta \right)}\sin\left(\beta \right)\cos\left(\alpha +\tan^{-1}\left(\frac{L\sin(\theta )}{L\cos\left(\theta \right)+d\cos\left(\phi_{i}\right)}\right)\right)+r+h\cos\left(\beta \right), \end{array}$$

(45) $$\begin{array}{cc} \; & \left|\right|n_{ij}\left|\right|=[L^{2}+r^{2}+d^{2}+h^{2}+2dL\cos\left(\theta \right)+2hr\cos\left(\beta \right)+2rd\cos\left(\alpha \right)\sin\left(\beta \right) \end{array}$$

(46) $$\begin{array}{cc} \; & +2rL\left(\cos\left(\alpha \right)\sin\left(\beta \right)\sin\left(\alpha \right)\sin\left(\beta \right)\right)){]}^{1/2} \end{array}$$

(47) $$\begin{array}{cc} \; & \approx \sqrt{L^{2}+d^{2}+2dL\cos\left(\theta \right)+r^{2}+h^{2}+2hr\cos\left(\beta \right)}. \end{array}$$

Since both the denominator and the numerator depend on θ, the solutions for the intervals, inverse cosine approximations, and modulus depend on θ. To address the dependency on θ, we use small Δθ steps to integrate and solve the approximations and modulus equations. The rate of change dependent on θ allows for nearly constant solutions in a certain θ interval. When we integrate small variances of θ, the sectors being integrated have their own probability density, which is

(48) $$\begin{array}{cc} \; & f_{L,\theta }^{\Delta \theta_{l}}=\frac{2L}{\Delta \theta_{l}R_{\Delta \theta_{l}}} \end{array}$$

(49) $$\begin{array}{cc} \; & R_{\Delta \theta_{l}}=\sum_{n=1}^{4}\frac{\Delta X_{n//2}^{2n+1}}{\tan(\theta_{l}^{-}-\left(n-1\right)\pi )}1_{\theta_{l}^{-}<\theta_{cr}^{n},\theta_{l}^{-}-\left(n-1\right)\pi )>0}+\frac{\Delta X_{n//2}^{2n}}{\cot(\frac{n\pi }{2}-\theta_{l}^{+})}1_{\theta_{l}^{+}>\theta_{cr}^{n},\frac{n\pi }{2}-\theta_{l}^{+}<\frac{\pi }{2}}, \end{array}$$

where $\theta_{l}^{-}$ corresponds to the lower bound of the interval and $\theta_{l}^{+}$ to the upper bound of the interval. $\Delta X_{n}^{k}$ corresponds to either $\Delta D_{n//2}$ or $\Delta Y_{n//2}$, while *k* refers to either the odd or even case. Using these intervals, we can rewrite the integral as

(50) $$\begin{array}{cc} \; & I_{tot}=\frac{1}{A}\sum_{l=1}^{N_{l}}P\left(\theta_{1j}\leq \Theta ,...,\theta_{N_{t}j}\leq \Theta |\theta \in \left[\theta_{l}^{-},\theta_{l}^{+}\right]\right), \end{array}$$

(51) $$\begin{array}{cc} \; & A=\sum_{l=1}^{N_{l}}0.5\Delta \theta_{l}R_{\Delta \theta_{l}}^{2}. \end{array}$$

The corresponding intervals generated for α are

(52) $$\begin{array}{cc} \; & \alpha \in \left[-\cos^{-1}\left(u_{d_{i}}\left(L,\theta \right)\right)-\tan^{-1}\left(\frac{L\sin(\alpha )}{L\cos\left(\alpha \right)+d_{i}}\right),\cos^{-1}\left(u_{d_{i}}\left(L,\theta \right)\right)-\tan^{-1}\left(\frac{L\sin(\alpha )}{L\cos\left(\alpha \right)+d_{i}}\right)\right], \end{array}$$

(53) $$\begin{array}{cc} \; & u_{d_{i}}\left(L,\theta \right)=\frac{\cos\left(\Theta \right)\sqrt{L^{2}+d^{2}+2dL\cos\left(\theta \right)+b^{2}}-a}{\sqrt{L^{2}+d^{2}+2dL\cos\left(\theta \right)}\sin\left(\beta \right)}. \end{array}$$

The interval shows that the modulus equations depend on θ. In the case of the threshold equations, they can be solved in a straightforward manner using the solutions given by the solutions for the d=0 case. Using these solutions, we can derive zero to four solutions for the threshold using

(54) $$\begin{array}{cc} \; & L_{m}^{\Delta \theta_{i}}=-d\cos\left(\theta_{i}^{avg}\right)\pm \sqrt{d\cos\left(\theta_{i}^{avg}\right)+d^{2}-L_{k}^{2}}, \end{array}$$

(55) $$\begin{array}{cc} \; & \theta_{i}^{avg}=\theta_{i}^{-}+0.5\Delta \theta_{i}. \end{array}$$

Since the solutions are centered around $-d\cos(\theta_{i}^{avg})$, we can use a similar method to generate the sequence for the case with d=0, using the reverse methodology for the case where $L<-d\cos(\theta_{i}^{avg})$. The new modulus breakpoints are

(56) $$\begin{array}{cc} \; & k_{1}=\cos^{2}\left(\Theta \right)-\sin^{2}\left(\beta \right)\cos^{2}\left(\theta_{l}^{avg}\right)\\ \; & k_{2}=\left(2d_{i}\cos\left(\Theta \right)+2a\sin\left(\beta \right)-2d_{i}\sin^{2}\left(\beta \right)\right)\cos\left(\theta_{l}^{avg}\right)\\ \; & k_{3}=d_{i}^{2}\cos^{2}\left(\Theta \right)\cos^{2}\left(theta_{l}^{avg}\right)+\cos^{2}\left(\Theta \right)\left(b^{2}+d_{i}^{2}+d_{i}^{2}\sin^{2}\left(\theta_{l}^{avg}\right)\right)-\\ \; & d_{i}^{2}\sin^{2}\left(\beta \right)-a^{2}+2ad_{i}\sin\left(\beta \right)\\ \; & B_{1,i,l}=\frac{k_{2}-\sqrt{k_{2}^{2}-4k_{1}k_{2}}}{2k_{1}}\\ \; & B_{2,i,l}=\frac{k_{2}+\sqrt{k_{2}^{2}-4k_{1}k_{2}}}{2k_{1}} \end{array}$$

Unlike in the SIMO case, the arrangement of the intervals does not follow a straightforward set of equations, as they are dependent on the corresponding phase which varies with both the distance *L* and the angle $\theta_{i}^{avg}$, which makes it possible, for example, that $B_{1,i,l}$ corresponds to a solution in the upper bound and $B_{2,i,l}$ corresponds to a solution in the lower bound.

Since tan−1(x) is injective from $-\frac{\pi }{2}$ to $\frac{\pi }{2}$, the function does not behave as desired between $\frac{\pi }{2}$ and $\frac{3\pi }{2}$. A solution to this is to patch the function using the following equation:

(57) $$\theta_{d_{i}}\left(L,\alpha \right)=\tan^{-1}\left(\frac{L\sin(\alpha )}{L\cos\left(\alpha \right)+d_{i}}\right)+\pi \left(1_{\theta_{i}^{avg}\in \left[\frac{\pi }{2},\frac{3\pi }{2}\right],L\geq -d\cos\left(\theta_{i}^{avg}\right),d_{i}\geq 0}+1_{L\geq -d\cos\left(\theta_{i}^{avg}\right),d_{i}\leq 0}\right).$$

Since we are assuming that all transmitters are on the same axis, we can assume that $d_{1}=0$ and $d_{n}\geq d_{1}$. Using this condition, we can divide the interval formation for a single transmitter in four cases depending on $\theta_{i}^{avg}$. It is also relevant to note that the breaking points, given the new equation, have up to four solutions. We use $B_{1,i,l}^{-},B_{2,i,l}^{-}$ for the cases where *L* is below the patching and $B_{1,i,l}^{+},B_{2,i,l}^{+}$ for the cases where *L* is above the patching. The solutions for the cases above and below the patching are in Appendix D.

Similarly to in the SIMO case, the resulting intervals have to be intersected to obtain the integrating intervals. Unlike in the SIMO case, the solution for the intersection does not have a closed, exact analytical solution. The equation can be solved using piecewise approximations or numerical methods. In this paper, we use the Newton–Raphson method to obtain both the minimum and maximum for each interval. A pair has a maximum of two real solutions, as the inverse of the tangent monotonically increases and the inverse cosine is either monotonically increasing on the positive side or has a single minimum. Thus, we can use both sides of the interval as starting points to obtain either zero, one, or two solutions. Given an interval from $L_{p+1}$ to $L_{p}$ of a set, *p*, such that every approximation of the inverse cosine is constant and no equation, $g_{d_{i}}^{+},g_{d_{i}}^{-}$, crosses π or −π, the resulting intervals are

(58) $$\begin{array}{cc} \; & \alpha \in \overset{\infty }{\underset{i=1}{⋃}}\left[g_{i1}^{-}1_{L\in [L_{i-1},L_{i}]},g_{i2}^{+}1_{L\in [L_{i-1},L_{i}]}\right]\cup \left[g_{i3}^{-}1_{L\in [L_{i-1},L_{i}]},g_{i4}^{+}1_{L\in [L_{i-1},L_{i}]}\right]\\ \; & g_{i1}=\max\left(g_{d_{1}}^{-},...,g_{d_{M}}^{-},-\pi \right),L\in \left[L_{i-1},L_{i}\right]\\ \; & g_{i2}=\min\left(g_{d_{1}}^{+},...,g_{d_{M+N}}^{+}\right),L\in \left[L_{i-1},L_{i}\right]\\ \; & g_{i3}=\max\left(g_{d_{1}}^{-},...,g_{d_{M+N}}^{-}\right),L\in \left[L_{i-1},L_{i}\right]\\ \; & g_{i4}=\min\left(g_{d_{1}}^{+},...,g_{d_{M}}^{+},\pi \right),L\in \left[L_{i-1},L_{i}\right] \end{array}$$

with *M* being the intervals that are continuous between −π and π and *N* the intervals that are not continuous. Unlike in the SIMO case, *M* can be zero under certain conditions. In this case, we define $g_{i1}$ and $g_{i4}$ as −π and π, respectively.

Using the succession of intervals χ corresponding to the intersections generated by the modulus equation, we obtain the expected probability as

(59) $$\begin{array}{cc} E(P\left(\theta_{1j}\leq \Theta ,...,\theta_{N_{r},j}\leq \Theta \right)) & =\sum_{n=1}^{\left|\chi \right|}\sum_{p=1}^{P_{n}}\sum_{m=1}^{M}[\int_{m\Delta \theta }^{(m+1)\Delta \theta }\int_{0}^{L_{max}}[\left(g_{p2}-g_{p1}\right)1_{L\in [L_{p-1},L_{p}]}\\ \; & +\left(g_{p4}-g_{p3}\right)1_{L\in [L_{p-1},L_{p}]})]f_{L,\theta }^{(m+0.5)\Delta \theta }dLd\theta ]\frac{1}{2\pi }d\alpha \end{array}$$

where *M* corresponds to the number of divisions. The solution of the integral for each bound is

(60) $$\begin{array}{cc} \int_{\theta }\int_{L}\int_{\alpha }\frac{g_{ip}}{2\pi }f_{L,\theta }^{\Delta \theta_{m}}dLd\theta d\alpha & =\frac{1}{2\pi \Delta \theta_{m}R_{\Delta \theta_{m}}}\int_{\theta }\int_{L}\int_{\alpha }2L(\cos^{-1}\left(u_{d_{ip}}\left(L,\theta \right)\right)-\\ \; & \tan-1(\frac{L\sin(\alpha )}{L\cos\left(\alpha \right)+d_{ip}})d\theta dLd\alpha .\\ \; & =\frac{1}{2\pi \Delta \theta_{m}R_{\Delta \theta_{m}}}\int_{\theta }\int_{L}\int_{\alpha }2L\\ \; & \left(\frac{a_{ip}}{\sin\beta }\frac{\cos\left(\Theta \right)\sqrt{u_{ip}^{2}+b^{2}}-a}{u_{ip}}+b_{ip}-\tan^{-1}\left(\frac{L\sin(\alpha )}{L\cos\left(\alpha \right)+d_{ip}}\right)\right)d\theta dLd\alpha .\\ \; & =\frac{1}{2\pi \Delta \theta_{m}R_{\Delta \theta_{m}}}\int_{\theta }\int_{L}\int_{\alpha }A_{ip}+B_{ip}-T_{ip}d\theta dLd\alpha \end{array}$$

where $A_{ip}$ corresponds to the terms associated with the coefficient $a_{ip}$ and $B_{ip}$ to those associated with the constant $b_{ip}$, and $T_{ip}$ is the integral of the inverse tangent. The integral of $A_{ip}$ can be approximated using first-order approximations of the square root, which results in

(61) $$\begin{array}{cc} A_{ip} & \approx \int_{m\Delta \theta }^{(m+1)\Delta \theta }\int_{L_{p-1}}^{L_{p}}(\frac{a_{ip}}{\sin(\beta )}(\cos\left(\Theta \right)(\left(1+\frac{b^{2}}{2u_{ip}^{2}}\right)1_{u_{ip}>b}\\ \; & +b\left(\frac{1}{u_{ip}}+\frac{u_{ip}}{2b^{2}}1_{u_{ip}<b}\right)-\frac{a}{u_{ip}}))LdLd\theta \\ \; & =A_{ip}^{1}+A_{ip}^{2}-A_{ip}^{3}, \end{array}$$

where the resulting expression is in Appendix C.

The expression for the integral of $B_{ip}$ is

(62) $$\begin{array}{c} B_{ip}=0.5\left(L_{p}^{2}-L_{p-1}^{2}\right)\Delta \theta \end{array}$$

On the other hand, the inverse tangent integral does not have a simple primitive when integrating by θ. Exploiting the fact that θ uses small steps, we linearize the inverse tangent around (m+0.5)Δθ. The resulting value is

(63) $$\begin{array}{cc} \; & T_{ip}^{L,\theta ,\phi }=\frac{L^{2}}{2}\left(\tan^{-1}\left(\frac{L\sin(\phi )}{L\cos\left(\phi \right)+d_{ip}}\right)\theta_{i}+\frac{Ld_{ip}\cos\left(\phi \right)+d_{ip}^{2}}{L^{2}+d_{ip}^{2}+2d_{ip}L\cos\left(\phi \right)}\left(\frac{\theta^{2}}{2}-\phi \theta \right)\right)\\ \; & +\frac{d_{ip}^{2}}{2}(\frac{\left(\sin(2\theta \right)}{2}(\tan^{-1}\left(\frac{d_{ip}\sin\left(\phi \right)}{d\cos(\phi )+L)}-\frac{Ld_{ip}\cos\left(\phi \right)+L^{2}}{L^{2}+d_{ip}^{2}+2d_{ip}L\cos\left(\phi \right)}\phi \right)\\ \; & +\frac{Ld_{ip}\cos\left(\phi \right)+L^{2}}{L^{2}+d_{ip}^{2}+2d_{ip}L\cos\left(\phi \right)}\left(\theta \frac{\sin(2\theta )}{2}+\frac{\cos(2\theta )}{4}\right)+\frac{Ld_{ip}}{2}\cos\left(\theta \right)+\\ \; & \frac{d_{ip}^{2}}{2}\left(\left(-\ln\left(L^{2}+d_{ip}^{2}\right)+1-\ln\left(2\right)\right)\frac{\cos(2\theta )}{4}-\sum_{n=1}^{\infty }{\left(\frac{2Ld_{ip}}{L^{2}+d_{ip}^{2}}\right)}^{n}{\left(-1\right)}^{n+1}\frac{\cos^{n+2}\left(\theta \right)}{n(n+2)}\right)\\ \; & T_{ip}=T_{ip}^{L_{p},\left(m+1\right)\Delta \theta ,\left(m+0.5\right)\Delta \theta }+T_{ip}^{L_{p-1},m\Delta \theta ,\left(m+0.5\right)\Delta \theta }-T_{ip}^{L_{p},m\Delta \theta ,\left(m+0.5\right)\Delta \theta }-\\ \; & T_{ip}^{L_{p-1},\left(m+1\right)\Delta \theta ,\left(m+0.5\right)\Delta \theta } \end{array}$$

## 7. Results and Discussions

**Figure 4:**
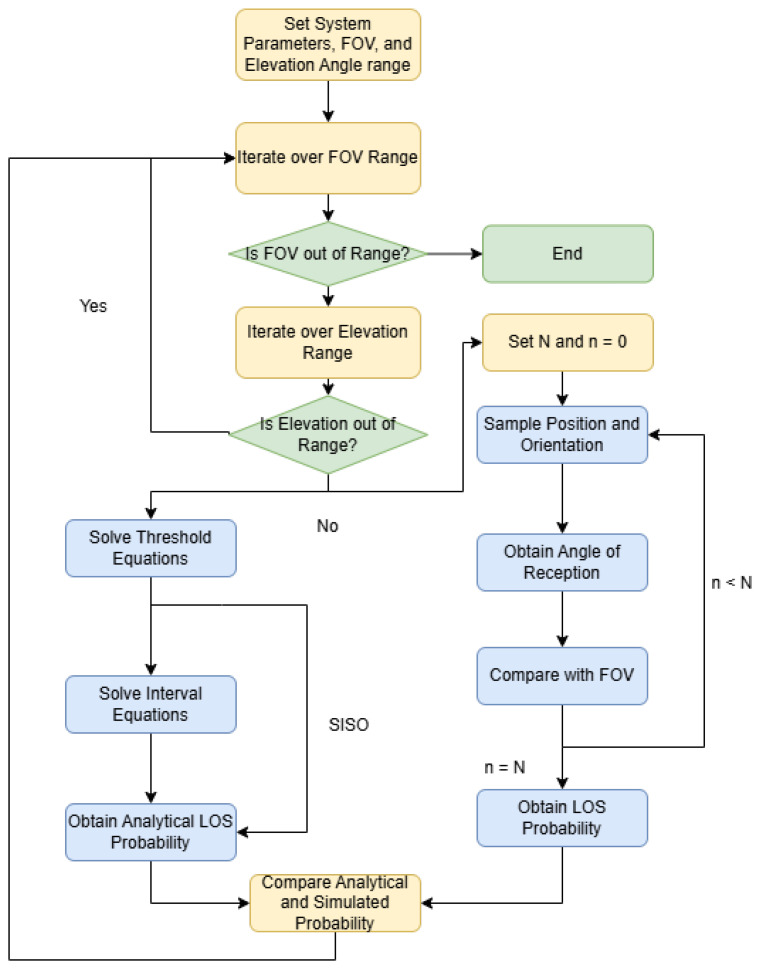
Flow chart of the methodology used to obtain the simulated and analytical results.

In this section, we present the numerical results for the LOS probability for each case. In each case, the user moves through a tunnel with a length of X=5[m] and a width of Y=3[m], and the main LED is placed at [1,1,3]. The radius of the helmet is considered to be r=0.05[m], and the height of the user is 1.8[m], so *h* is 1.2[m]. We computed the simulated results using Monte Carlo sampling over the user position and orientation, which allowed us to obtain the receiving angle and compare it with the FOV. We obtained the results using a variable β for each FOV Θ, since a higher field of view would trivially imply a higher LoS probability. Figure 4 shows the methodology used to obtain the simulated and analytical results.

### 7.1. SISO Case

**Figure 5:**
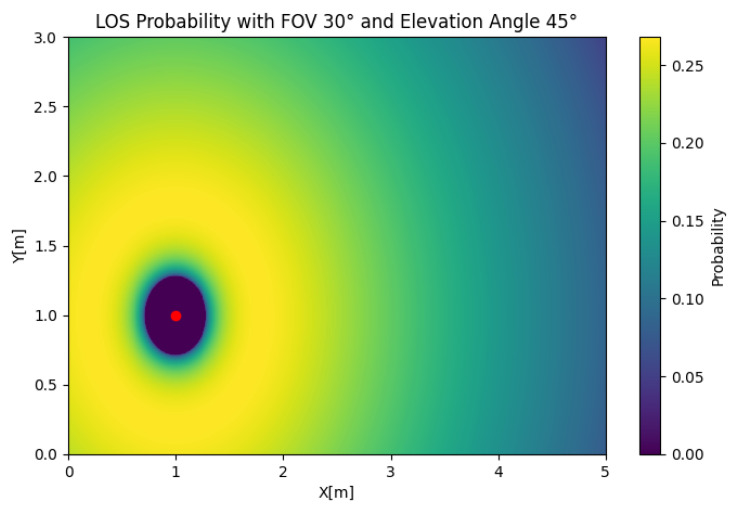
SISO LOS probability with Θ=30 and β=45.

**Figure 6:**
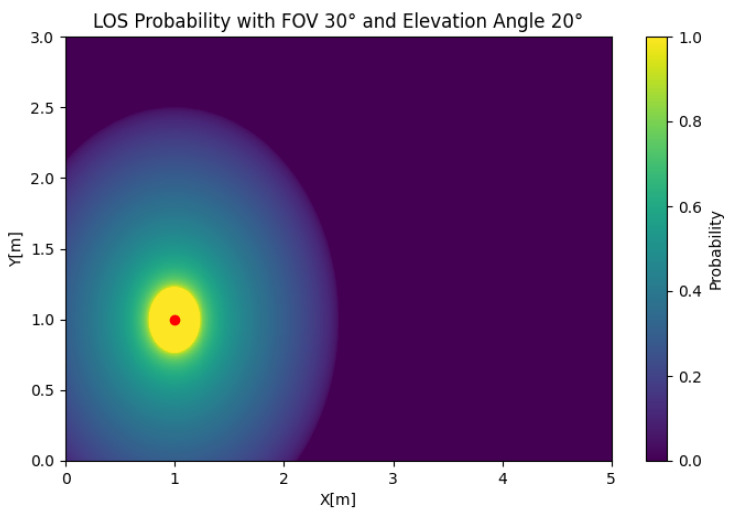
SISO LOS probability with Θ=30 and β=20.

In Figure 5, the SISO LOS probability is depicted for any position of the user. In this case, we took Θ=30 and β=45 to illustrate the effect of the position of the user on the LOS probability. The figure shows that the LOS probability was 0 around the LED, which was a product of the value of β, which oriented the receiver away from the source. However, if we set β=20, as shown in Figure 6, the LOS probability rapidly decayed with respect to the radius. This behavior created a trade-off in the orientation, where orientations closer to the orthogonal case could be suboptimal in certain setups.

**Figure 7:**
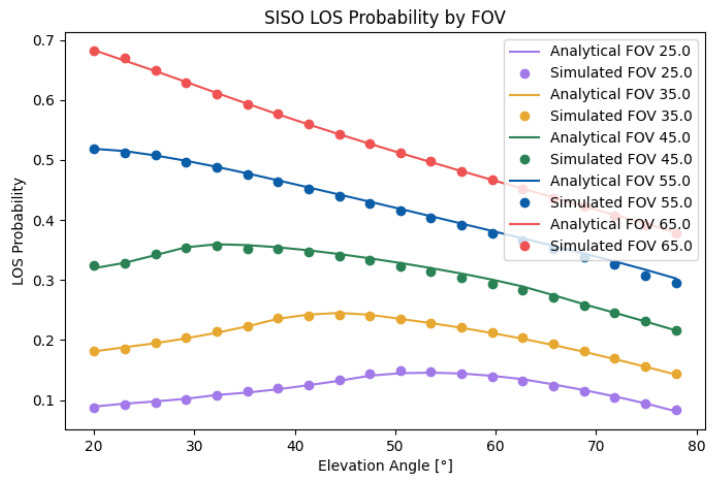
Analytical and simulated SISO LOS probabilities.

In Figure 7, the analytical SISO case is compared with the simulated case. As can be seen, the analytical case matched perfectly with the simulated data, with an error of 1.15%. Evidently, the LoS probability increased monotonously with respect to the FOV. However, each FOV had an optimal elevation angle, which decreased with the FOV. The optimal value was not only a product of β but also a product of *h* and *r*. A higher *h* increased the probability, with the cost of a higher path loss. For example, in the case of an FOV of $30^{\circ }$, increasing the height difference from 1.2[m] to 1.6[m] increased the LoS probability by 0.04, or 7%.

### 7.2. SIMO Case

**Figure 8:**
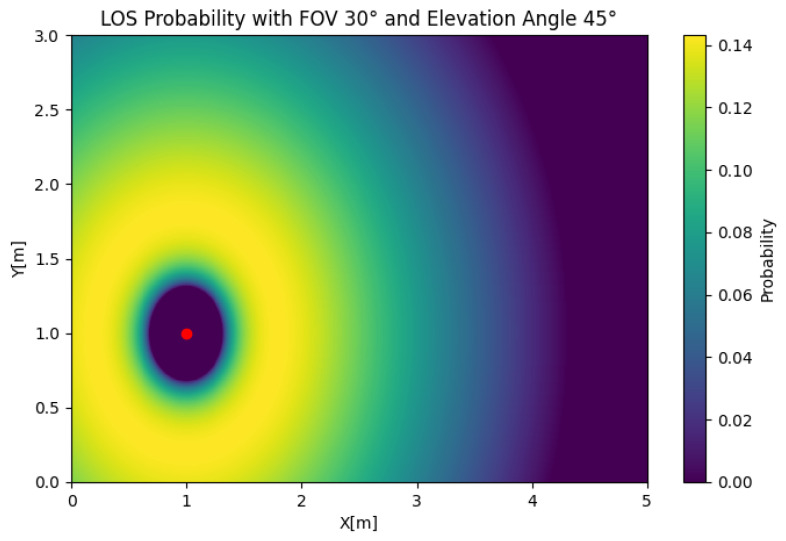
SISO LOS probability with Θ=30, β=45, and $\alpha_{1}=90$.

**Figure 9:**
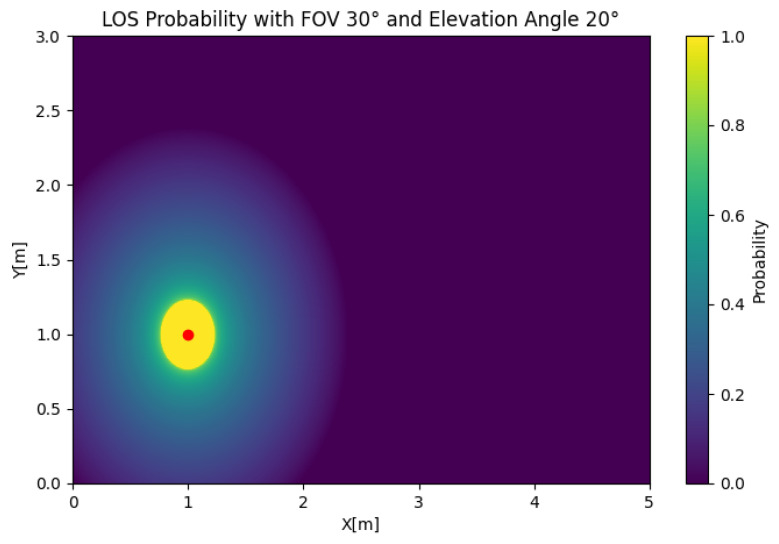
SISO LOS probability with Θ=30, β=20, and $\alpha_{1}=90$.

In Figure 8, the SIMO LOS probability is depicted for any position of the user. To reduce the complexity of the problem, we obtained the results using two receivers. In this case, we took an FOV of Θ=30, an elevation of β=45, and a displacement of $\alpha_{1}=90$ to illustrate the effect of the user position on the LoS probability. The figure shows that the LOS probability was 0 around the LED, which was a product of the value of β, which oriented both receivers away from the source. We could also observe that the LOS probability decayed faster than in the SISO case with a similar setup, which demonstrates its sensitivity to an orientation away from the source. If we set β=20, as shown in Figure 9, the LOS probability rapidly decayed with respect to the radius. However, the reduction in the total LOS probability was comparatively not as strong as in the SISO case, which was explained by the lack of coverage in the outer areas of the cell for the β=45 case.

**Figure 10:**
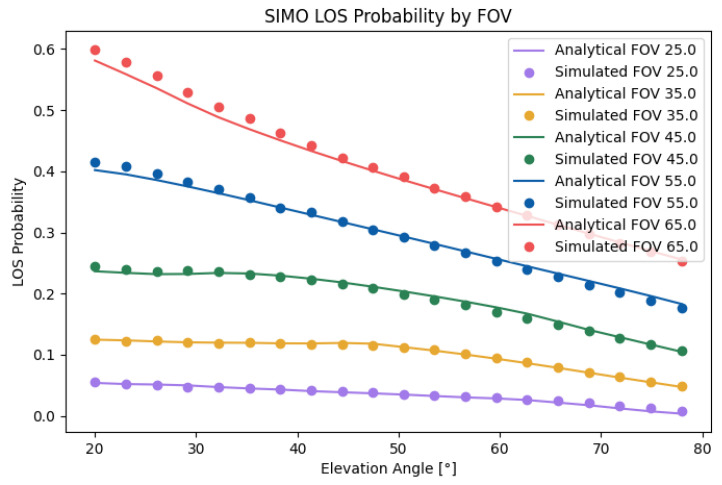
Analytical and simulated SIMO LOS probabilities with $\alpha_{1}=90$.

Figure 10 compares the analytical results with the simulated results for the SIMO case. As can be seen, the analytical results matched well with the simulated results. The small error with a higher FOV was a product of the effect of the approximations of the inverse cosine function. The total error was 2.32%. Unlike in the SISO case, the LOS probability was rather constant with low elevation angles until it started falling off from the same peak as in the SISO case. This effect can be attributed to the faster fall in the LoS probability with respect to the radius. As the effect was a product of the decay near the borders, placing the transmitter closer to the center would heavily improve the LOS probability inside the cell for higher elevation angles while not impacting the lower elevation angles. In the calculated scenario, moving the transmitter from [1,1] to [1,1.5] improved the LoS probability from 0.095 to 0.103 in cases with a high elevation angle, corresponding to an increase of 3%. Similarly to in the SISO case, the optimal elevation was a factor of both the elevation and height. Increasing the height difference from 1.2[m] to 1.6[m] increased the probability by 0.45, or 11%.

### 7.3. MISO Case

**Figure 11:**
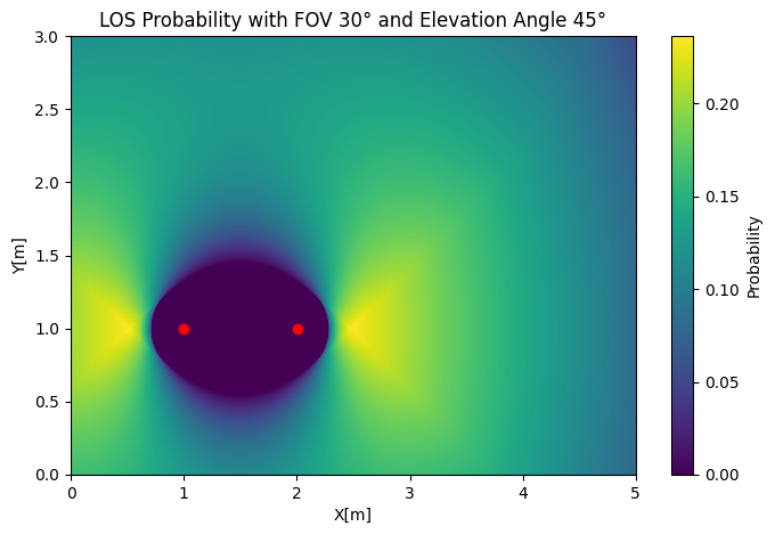
MISO LOS probability with Θ=30, β=45, and d=1.

**Figure 12:**
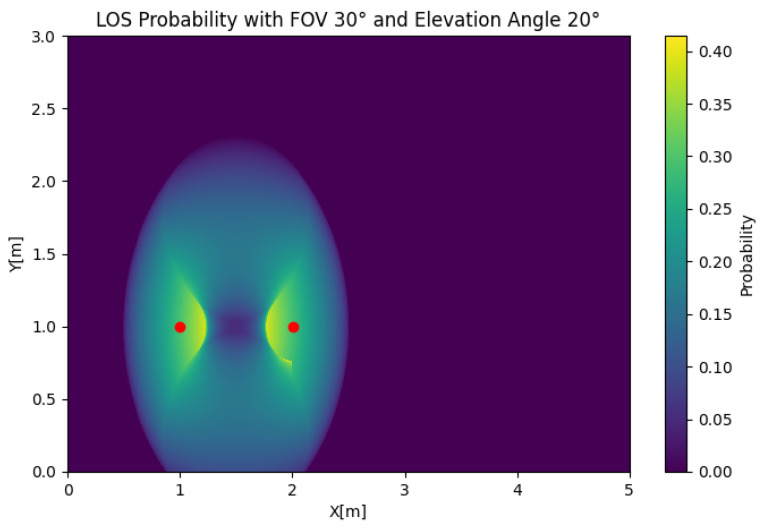
MISO LOS probability with Θ=30, β=20, and d=1.

In Figure 11, the MISO LoS probability is depicted for any position of the user. To reduce the complexity of the problem, we obtained the results using two transmitters. In this case, we took an FOV of Θ=30 and an elevation of β=45 and used two transmitters that were d=1[m] from each other. The figure shows that the LOS probability was 0 around both LEDs, which was the product of combining the effect of the value of β orienting both receivers away from one of the sources and the orientation being exclusively towards one of the transmitters. We could also observe that the LOS probability had a maximum at the sides of the transmitter, creating two lobes that decreased with respect to the radius, which was an important difference compared to the circular contours obtained in the SIMO and SISO cases. If we set β=20, as shown in Figure 12, the LOS probability had the same symmetric shape, but the maximum probability was obtained between the LEDs. In the MISO case, the change in the LOS probability due to the elevation was significant. However, a reduced simultaneous LOS probability can be advantageous, depending on the desired properties of the system.

**Figure 13:**
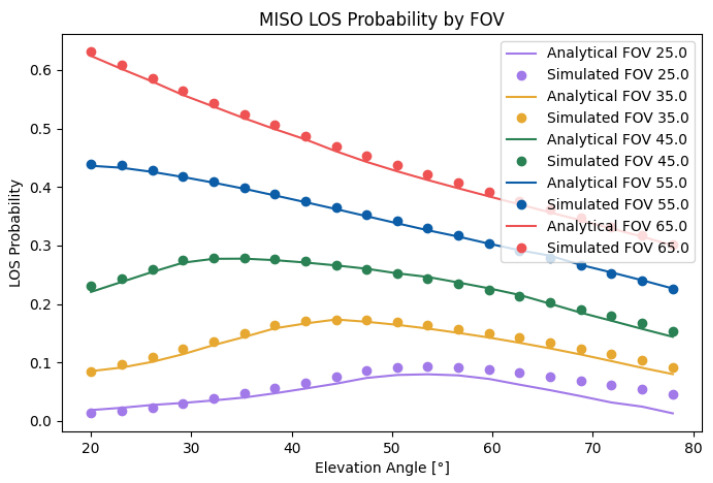
Analytical and simulated MISO LOS probabilities with d=1.

Figure 13 compares the analytical results with the simulated results for the SIMO case. As can be seen, the analytical results matched well with the simulated results. The small error with a lower FOV was a product of the effect of the approximations of the argument of the inverse cosine function. The total error was 2.52%. Similarly to in the SISO case, the LOS probability had a clear maximum dependent on the FOV. The peak coincided approximately with that in the SISO case, but it depended on both *d* and *h*. The MISO probability followed similar behavior to the SISO probability because the former can be understood as the intersection of the latter, while the SIMO probability followed different behavior given the reduced effect of the relative orientation.

## 8. Conclusions

In this paper, we obtained a generalized analytical expression for the LOS probability for SISO, SIMO, and MISO cases when the probability density function of the reception angle was a function of the receiver’s geometry and the user’s mobility. The expressions showed a close alignment between the simulation and analytical results, with slight errors produced by the approximations necessary to obtain an analytical integration form. The error of the analytical expression was 1.15%, 2.32%, and 2.52% for the SISO, SIMO, and MISO cases, respectively. We studied the effect of the receiver’s angular elevation with a constant FOV, which showed that an optimal elevation existed for each FOV. Furthermore, the optimality of this angular elevation was not preserved from the SISO case to the SIMO or MISO cases. The effect of the height on the LoS probability was also obtained, showing that higher height differences increased the LoS probability. Finally, the resulting analytical expressions are a representative approach to the application of the underground mining proposal, showing predictive power to optimize the channel with respect to the geometrical elements inside it.

The SIMO and MISO analytical expressions can be expanded to an MIMO case, which can be performed in future work. Furthermore, future work will involve a comparison between different communication schemes and prove the improvements in an experimental setup.

## Figures and Tables

**Table 1:** Scenario parameters.

Parameters	Scenario
**Tunnel:**	
Length, X (m)	6
Width, Y (m)	3
Height, Z (m)	3.5
Wall reflection coefficient, ρ	0.6
Wall rotation angle, $\alpha_{w}$	U [0, 180]
Wall tilt angle, $\beta_{w}$	U [0, 180]
Scatterer reflection coefficient, $\rho_{s}$	0.1
Number of scatterers, N	40
**Transmitter:**	
Position 1, (x, y, z) (m)	(1, 0.5, 3)
Position 1, (x, y, z) (m)	(2, 0.5, 3)
Transmitter rotation angle, $\alpha_{i}$	0
Transmitter tilt angle, $\beta_{i}$	45
**Receiver:**	
Receiver tilt angle, $\alpha_{j}$	55
FOV, Θ	55
Radius, (m)	0.1
FOV, Θ	$55^{\circ }$
**Shadowing:**	
Width probability density, $g_{v}\left(w\right)$	N (2, 0.5)
Height probability density, $g_{v}\left(h\right)$	N (1.5, 0.5)
Y probability density, $f_{v}\left(y\right)$	U [0, 3]
X probability density, $f_{v}\left(x\right)$	U [0, 5]
**Resolution:**	
Time resolution (ns)	0.25
Area elements in X	18
Area elements in Y	18
Area elements in Z	15
Spatial resolution in X (m)	0.1
Spatial resolution in Y (m)	0.1
Spatial resolution in Z (m)	0.1

**Table 2:** System parameters.

Parameters	Values
Absolute temperature, $T_{k}\left(K\right)$	295 [20]
Atmospheric parameter, γ	0.017 [21]
Atmospheric parameter, *g*	0.72 [21]
Atmospheric parameter, *f*	0.5 [21]
Average transmitted power, $P_{i}\left(W\right)$	1 [22]
Background dark current, $I_{bg}$ (nA)	10 [20]
Band-pass filter of transmission	1 [23]
Boltzmann constant, κ(J/K)	$1.38\times 10^{-23}$ [20]
Capacitance, $C_{pd}$	$1.12\times 10^{-8}$ [20]
Radius area, $R_{r}$	1 [24]
Electronic charge, q(C)	$1.6\times 10^{-19}$ [20]
FET channel noise factor, Γ	1.5 [20]
FET trans-conductance, $g_{m}\left(S\right)$	0.03 [20]
Optical gain, $g(\dot{)}$	1 [25]
Lambertian mode number, *m*	1 [21]
Mie scattering coefficient, $k_{m}$	U [0, 10] [21]
Noise bandwidth factor, $I_{2}$	0.562 [20]
Noise bandwidth factor, $I_{3}$	0.0868 [20]
Open-loop voltage gain, *G*	10 [20]
Physical active area, $A_{p}\left(cm^{2}\right)$	1 [23]
Rayleigh scattering coefficient, $k_{r}$	U [0.01, 0.1] [24]
Refractive index, η	1.5 [23]
Responsivity, $R_{PD}\left(A/W\right)$	0.53 [23]
Half-power semi-angle, $\Phi_{1/2}$	60 [26]
Mie’s density, *N*	$2\times 10^{6}$ [27]
Wavelength, λ	500 [nm] [27]
Particle size, ρ	$1\times 10^{-5}$ [m] [27]
Refractive coefficient, *m*	1.5 + j0.0014
Rayleigh’s density, $N_{s}$	$2.547\times 10^{19}$ [28]
Depolarization factor, δ	$2.547\times 10^{-19}$ [28]

**Figure A1:**
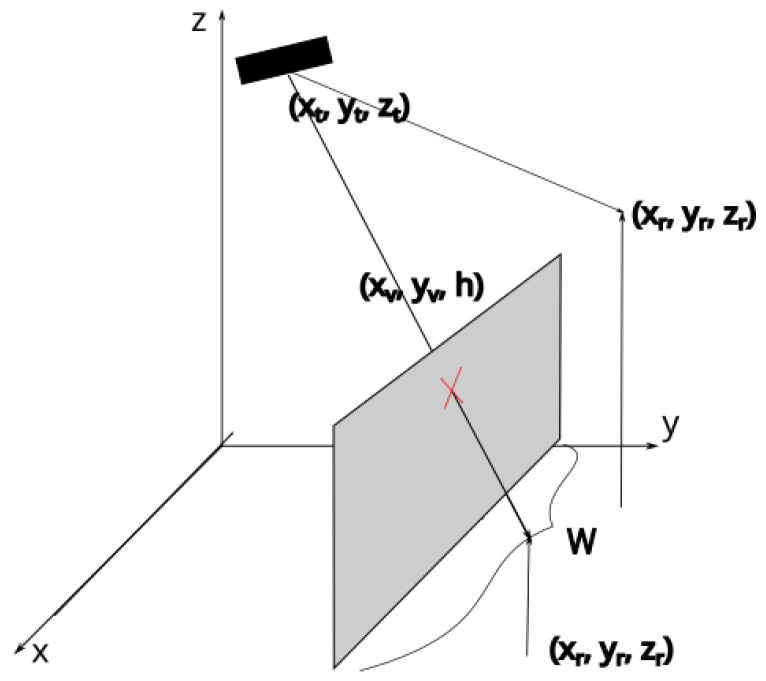
The described scenario, where $x_{v}$ and $y_{v}$ are the coordinates of the object, *h* is its height, and *w* is its width along the *X* axis.

## Data Availability

The dataset is available on request from the authors.

## Appendix A

The integral that defines the probability of $p_{v}$ can be described by the following expression:

(A1) $$E\left(p_{v}\right)=\int_{0}^{X}\int_{0}^{Y}\left(\underset{w\geq 2d\left(x_{v},y_{v}\right),h\geq s\left(x_{v},y_{v}\right)}{\;\int \int \;}g\left(w,h\right)dwdh\right)f\left(x,y\right)dydx,$$

where *X* and *Y* are the dimensions of the tunnel in the horizontal and vertical axes, respectively. The density probability of the width and height of the object is defined by g(w,h), and the density probability of the object position is defined by f(x,y). Nevertheless, computing this integral requires substantial effort. Thus, using smoothing, we have the following expression:

(A2) $$E\left(p_{v}\right)=E\left(E\left(p_{v}|w,h\right)\right),$$

where $E(p_{v}|w,h)$ simplifies to

(A3) $$E\left(p_{v}|w,h\right)=\int_{0}^{X}\int_{0}^{Y}1_{w\geq 2d\left(x_{v},y_{v}\right),h\geq s\left(x_{v},y_{v}\right)}f\left(x,y\right)dydx.$$

The limits of the unit value are derived from the presence of an obstruction, which is mathematically described by the function d(x,y) and the function s(x,y), based on the geometry provided in Figure A1. The function d(x,y) determines whether an obstruction is produced in the *X* axis, which can be determined using the distance from the center of the object to the projection on the ground of the signal sent between the receiver and the transmitter. The function s(x,y) determines whether an obstruction is produced in the *Z* axis. The expression of d(x,y) for a transmitter and receiver pair is

(A4) $$d{\left(x_{v},y_{v}\right)}^{i,j}=\frac{|\left(y_{i}^{T}-y_{j}^{R}\right)x_{v}-\left(x_{i}^{T}-x_{j}^{R}\right)y_{v}-x_{j}^{R}y_{i}^{T}+x_{i}^{T}y_{j}^{R}|}{\sqrt{{\left(y_{i}^{T}-y_{j}^{R}\right)}^{2}+{\left(x_{i}^{T}-x_{j}^{R}\right)}^{2}}},$$

where $x_{i}^{T},y_{i}^{T}$ are the coordinates of the transmitter, $x_{j}^{R},y_{j}^{R}$ are the coordinates of the receiver, and $x_{v},y_{v}$ are the coordinates of the center of the obstacle.

The expression of s(x,y) is a modified version of the one in [18], since symmetry is desired along the *X* axis. The function is

(A5) $$\begin{array}{cc} s{\left(x_{v},y_{v}\right)}^{i,j} & =\frac{{\left(y_{i}^{T}-y_{j}^{R}\right)}^{2}+{\left(x_{i}^{T}-x_{j}^{R}\right)}^{2}}{2\sqrt{{\left(y_{i}^{T}-y_{j}^{R}\right)}^{2}+{\left(x_{i}^{T}-x_{j}^{R}\right)}^{2}}}\\ \; & +\frac{{\left(\frac{\left(y_{v}-y_{j}R\right)}{\alpha }\right)}^{2}+{\left(y_{v}-y_{j}^{R}\right)}^{2}}{2\sqrt{{\left(y_{i}^{T}-y_{j}^{R}\right)}^{2}+{\left(x_{i}^{T}-x_{j}^{R}\right)}^{2}}}\\ \; & -\frac{[{\left(\frac{\left(y_{v}-y_{i}R\right)}{\alpha }\right)}^{2}+{\left(y_{v}-y_{i}^{T}\right)}^{2}}{2\sqrt{{\left(y_{i}^{T}-y_{j}^{R}\right)}^{2}+{\left(x_{i}^{T}-x_{j}^{R}\right)}^{2}}}+z_{j}^{R}, \end{array}$$

where α is the slope of the obstacle obstruction:

(A6) $$\alpha =\frac{y_{i}-y_{j}}{x_{i}-x_{j}}$$

Furthermore, we can rewrite the integral $E(p_{v}|w,h)$ as an equivalent to the sum

(A7) $$E\left(p_{v}|w,h\right)=\sum_{i=1}^{4}\sum_{j=1}^{2}I_{ij}-\sum_{i=1}^{4}\sum_{j=3}^{4}I_{ij},$$

where each $I_{ij}$ is defined by being part of a certain subset of geometrical conditions.

The integral of $E(p_{v}|w,h)$ can be divided into four cases. These four cases arise from different values of d(x,y) and s(x,y). The first two cases come from assuming

(A8) $$\left(y_{i}^{T}-y_{j}^{R}\right)x_{v}-\left(x_{i}^{T}-x_{j}^{R}\right)y_{v}-x_{j}^{R}y_{i}^{T}+x_{i}^{T}y_{j}^{R}\geq 0.$$

In this case, the inequality

(A9) $$d{\left(x_{v},y_{v}\right)}^{i,j}\leq \frac{w}{2},$$

transforms into the following inequality:

(A10) $$\begin{array}{c} \frac{y_{ij}}{x_{i}j}x_{v}+\frac{\left(x_{i}y_{j}-x_{j}y_{i}\right)}{x_{ij}}-\frac{wd_{ij}}{2}\leq y_{v}, \end{array}$$

(A11) $$\begin{array}{c} \alpha x_{v}+W_{1}-W_{2}\leq y_{v}. \end{array}$$

Since the inequality holds, we also have the following inequality:

(A12) $$\alpha x_{v}+W_{1}\geq y_{v}.$$

In this case, $x_{ij}\geq 0$, d≥0, and α≤0. The second case occurs if $x_{ij}\leq 0$. Since the sign is negative, the inequalities are expressed as

(A13) $$\begin{array}{c} \alpha x_{v}+W_{1}\leq y_{v}, \end{array}$$

(A14) $$\begin{array}{c} \alpha x_{v}+W_{1}-W_{2}\geq y_{v}. \end{array}$$

The third and fourth cases follow the same logic but with d≤0. In the third case, $x_{ij}\geq 0$, so the inequalities are

(A15) $$\begin{array}{c} \alpha x_{v}+W_{1}\leq y_{v}, \end{array}$$

(A16) $$\begin{array}{c} \alpha x_{v}+W_{1}+W_{2}\geq y_{v}. \end{array}$$

In the fourth case, $x_{ij}\leq 0$, so the inequalities are

(A17) $$\begin{array}{c} \alpha x_{v}+W_{1}\geq y_{v}, \end{array}$$

(A18) $$\begin{array}{c} \alpha x_{v}+W_{1}+W_{2}\leq y_{v}. \end{array}$$

All these inequalities are conditions for an obstruction along the *X* axis. In the case of the *Z* axis, we have one condition, which is

(A19) $$\begin{array}{c} \frac{h-h_{j}d_{ij}}{\cot\delta (1+\frac{1}{\alpha^{2}})}-\left(d_{i}j^{2}+2y_{v}y_{ij}-y_{j}^{2}+y_{i}^{2}\right)\leq y_{v}, \end{array}$$

(A20) $$\begin{array}{c} H_{1}-H_{2}\leq y_{v}. \end{array}$$

We integrate over the *Y* axis using

(A21) $$I_{y}=\int_{y_{min}}^{y_{max}}1_{w\geq 2d\left(x_{v},y_{v}\right),h\geq s\left(x_{v},y_{v}\right)}f\left(x,y\right)dy.$$

with $f\left(x,y\right)=\frac{1}{XY}$ being a uniform distribution. For each case, the integral will take a different form. The four integrals over *Y* are

$$\begin{array}{cc} \; & I_{1}^{y}=1_{\alpha x_{v}+W_{1}\geq y_{min}}1_{\alpha x_{v}+W_{1}-W_{2}\leq y_{max}}1_{H_{1}-H_{2}\leq y_{max}}\\ \; & (\min\left(y_{max},\alpha x_{v}+W_{1}\right)-\max\left(y_{min},H_{1}-H_{2},\alpha x_{v}+W_{1}-W_{2}\right)),\\ \; & I_{2}^{y}=1_{\alpha x_{v}+W_{1}\leq y_{min}}1_{\alpha x_{v}+W_{1}-W_{2}\geq y_{max}}1_{H_{1}-H_{2}\leq y_{max}}\\ \; & (\min\left(y_{max},\alpha x_{v}+W_{1}-W_{2}\right)-\max\left(y_{min},H_{1}-H_{2},\alpha x_{v}+W_{1}\right)),\\ \; & I_{3}^{y}=1_{\alpha x_{v}+W_{1}\leq y_{min}}1_{\alpha x_{v}+W_{1}+W_{2}\geq y_{max}}1_{H_{1}-H_{2}\leq y_{max}}\\ \; & (\min\left(y_{max},\alpha x_{v}+W_{1}+W_{2}\right)-\max\left(y_{min},H_{1}-H_{2},\alpha x_{v}+W_{1}\right)),\\ \; & I_{4}^{y}=1_{\alpha x_{v}+W_{1}\geq y_{min}}1_{\alpha x_{v}+W_{1}+W_{2}\leq y_{max}}1_{H_{1}-H_{2}\leq y_{max}}\\ \; & (\min\left(y_{max},\alpha x_{v}+W_{1}\right)-\max\left(y_{min},H_{1}-H_{2},\alpha x_{v}+W_{1}+W_{2}\right)). \end{array}$$

Letting $\gamma =\max(y_{m}in,H_{1}-H_{2})$, we obtain the indexes of the integrals of the sum $\sum \sum I_{ij}$. For the integral $I_{1}^{Y}$, we have

$$\begin{array}{cc} \; & I_{11}=y_{max}\left(\min\left(x_{max},\frac{\gamma -W_{1}}{\alpha },\frac{y_{m}ax-W_{1}}{\alpha }\right)-\max\left(0,\frac{y_{max}+W_{2}-W_{1}}{\alpha }\right)\right)\\ \; & 1_{0\leq (\gamma -W_{1})}1_{x_{max}\geq \frac{y_{max}+W_{2}-W_{1}}{\alpha }}1_{0\leq y_{max}-W_{1}}1_{H_{1}-H_{2}\leq y_{max}},\\ \; & I_{12}=\alpha \frac{x^{2}}{2}+W_{1}{|}_{lb_{12},ub_{12}}1_{0\leq (\gamma -W_{1})}1_{x_{max}\geq \frac{y_{max}+W_{2}-W_{1}}{\alpha }}1_{x_{max}\geq y_{max}-W_{1}}1_{H_{1}-H_{2}\leq y_{max}},\\ \; & I_{13}=\gamma \left(\min\left(x_{max},\frac{y_{m}in-W_{1}}{\alpha }\right)-\max\left(0,\frac{\left(y_{max}+W_{2}-W_{1}\right)}{\alpha },\frac{\gamma +W_{2}-W_{1}}{\alpha }\right)\right)\\ \; & 1_{0\leq (\gamma -W_{1})}1_{x_{max}\geq \frac{y_{max}+W_{2}-W_{1}}{\alpha }}1_{x_{max}\geq \frac{\gamma +W_{2}-W_{1}}{\alpha }}1_{H_{1}-H_{2}\leq y_{max}},\\ \; & I_{14}=\alpha \frac{x^{2}}{2}+\left(W_{1}-W_{2}\right)x{|}_{lb_{14},ub_{14}}1_{0\leq (\gamma -W_{1})}1_{x_{max}\geq \frac{y_{max}+W_{2}-W_{1}}{\alpha }}1_{0\leq \frac{\gamma +W_{2}-W_{1}}{\alpha }}1_{H_{1}-H_{2}\leq y_{max}},\\ \; & ub_{12}=\min\left(x_{max},\frac{y_{m}in-W_{1}}{\alpha }\right),\\ \; & lb_{12}=\max\left(0,\frac{y_{max}+W_{2}-W_{1}}{\alpha },\frac{y_{m}ax-W_{1}}{\alpha }\right),\\ \; & ub_{14}=\min\left(x_{max},\frac{y_{min}-W_{1}}{\alpha },\frac{\gamma +W_{2}-W_{1}}{\alpha }\right),\\ \; & lb_{14}=\max\left(0,\frac{y_{max}+W_{2}-W_{1}}{\alpha }\right). \end{array}$$

For the integral $I_{2}^{Y}$, we have

$$\begin{array}{cc} \; & I_{21}=y_{max}\left(\min\left(x_{max},\frac{y_{max}-W_{1}}{\alpha }\right)-\max\left(0,\frac{y_{max}+W_{2}-W_{1}}{\alpha },\frac{\gamma +W_{2}-W_{1}}{\alpha }\right)\right)\\ \; & 1_{0\leq (y_{max}-W_{1})}1_{x_{max}\geq \frac{\gamma +W_{2}-W_{1}}{\alpha }}1_{x_{max}\geq \frac{y_{max}+W_{2}-W_{1}}{\alpha }}1_{H_{1}-H_{2}\leq y_{max}},\\ \; & I_{22}=\alpha \frac{x^{2}}{2}+\left(W_{1}-W_{2}\right)x{|}_{lb_{22},ub_{22}}1_{0\leq (y_{max}-W_{1})}1_{x_{max}\geq \frac{\gamma +W_{2}-W_{1}}{\alpha }}1_{0\leq \frac{y_{max}-W_{2}-W_{1}}{\alpha }}1_{H_{1}-H_{2}\leq y_{max}},\\ \; & I_{23}=\gamma \left(\min\left(x_{max},\frac{y_{m}ax-W_{1}}{\alpha },\frac{\gamma -W_{1}}{\alpha }\right)-\max\left(0,\frac{\left(\gamma +W_{2}-W_{1}\right)}{\alpha }\right)\right)\\ \; & 1_{0\leq (\gamma -W_{1})}1_{0\leq \frac{\gamma -W_{1}}{\alpha }}1_{x_{max}\geq \frac{\gamma +W_{2}-W_{1}}{\alpha }}1_{H_{1}-H_{2}\leq y_{max}},\\ \; & I_{24}=\alpha \frac{x^{2}}{2}+W_{1}x{|}_{lb_{24},ub_{24}}1_{0\leq (y_{max}-W_{1})}1_{x_{max}\geq \frac{\gamma -W_{1}}{\alpha }}1_{x_{max}\geq \frac{\gamma +W_{2}-W_{1}}{\alpha }}1_{H_{1}-H_{2}\leq y_{max}},\\ \; & ub_{22}=\min\left(x_{max},\frac{y_{max}-W_{1}}{\alpha },\frac{y_{max}+W_{2}-W_{1}}{\alpha }\right),\\ \; & lb_{22}=\max\left(0,\frac{\gamma +W_{2}-W_{1}}{\alpha }\right),\\ \; & ub_{24}=\min\left(x_{max},\frac{\gamma -W_{1}}{\alpha }\right),\\ \; & lb_{24}=\max\left(0,\frac{\gamma +W_{2}-W_{1}}{\alpha },\frac{\gamma -W_{1}}{\alpha }\right). \end{array}$$

For the integral $I_{3}^{Y}$, we have

$$\begin{array}{cc} \; & I_{31}=y_{max}\left(\min\left(x_{max},\frac{\gamma -W_{2}-W_{1}}{\alpha },\frac{y_{max}-W_{2}-W_{1}}{\alpha }\right)-\max\left(0,\frac{y_{max}-W_{1}}{\alpha }\right)\right)\\ \; & 1_{x_{max}\geq \frac{\left(y_{max}-W_{1}\right)}{\alpha }}1_{0\leq \frac{\gamma -W_{2}-W_{1}}{\alpha }}1_{0\leq \frac{y_{max}-W_{2}-W_{1}}{\alpha }}1_{H_{1}-H_{2}\leq y_{max}},\\ \; & I_{32}=\alpha \frac{x^{2}}{2}+\left(W_{1}+W_{2}\right)x{|}_{lb_{32},ub_{32}}1_{0\leq (\gamma -W_{2}-W_{1})}1_{x_{max}\geq \frac{y_{max}-W_{1}}{\alpha }}1_{x_{max}\geq \frac{y_{max}-W_{2}-W_{1}}{\alpha }}1_{H_{1}-H_{2}\leq y_{max}},\\ \; & I_{33}=\gamma \left(\min\left(x_{max},\frac{y_{m}ax-W_{1}-W_{2}}{\alpha }\right)-\max\left(0,\frac{\left(y_{max}-W_{1}\right)}{\alpha },\frac{\gamma -W_{1}}{\alpha }\right)\right)\\ \; & 1_{0\leq (\gamma -W_{1}-W_{2})}1_{x_{max}\geq \frac{\gamma -W_{1}}{\alpha }}1_{x_{max}\geq \frac{y_{max}-W_{1}}{\alpha }}1_{H_{1}-H_{2}\leq y_{max}},\\ \; & I_{34}=\alpha \frac{x^{2}}{2}+W_{1}x{|}_{lb_{34},ub_{34}}1_{0\leq (\gamma -W_{1}-W_{2})}1_{x_{max}\geq \frac{\gamma -W_{1}}{\alpha }}1_{0\leq \frac{\gamma -W_{1}}{\alpha }}1_{H_{1}-H_{2}\leq y_{max}},\\ \; & ub_{32}=\min\left(x_{max},\frac{y_{max}+W_{2}-W_{1}}{\alpha }\right),\\ \; & lb_{32}=\max\left(0,\frac{\gamma -W_{2}-W_{1}}{\alpha },\frac{y_{max}-W_{1}}{\alpha }\right),\\ \; & ub_{34}=\min\left(x_{max},\frac{\gamma -W_{2}-W_{1}}{\alpha },\frac{\gamma -W_{1}}{\alpha }\right),\\ \; & lb_{34}=\max\left(0,\frac{y_{max}-W_{1}}{\alpha }\right). \end{array}$$

Finally, for the integral $I_{4}^{Y}$, we have

$$\begin{array}{cc} \; & I_{41}=y_{max}(\min\left(x_{max},\frac{y_{max}-W_{2}-W_{1}}{\alpha }\right)-\max\left(0,\frac{y_{max}-W_{1}}{\alpha },\frac{\gamma -W_{1}}{\alpha }\right)\\ \; & 1_{x_{max}\geq \frac{\left(\gamma -W_{1}\right)}{\alpha }}1_{0\leq \frac{y_{max}-W_{2}-W_{1}}{\alpha }}1_{x_{max}\geq \frac{y_{max}-W_{1}}{\alpha }}1_{H_{1}-H_{2}\leq y_{max}},\\ \; & I_{42}=\alpha \frac{x^{2}}{2}+\left(W_{1}\right)x{|}_{lb_{42},ub_{42}}1_{0\leq (y_{max}-W_{1})}1_{x_{max}\geq \frac{\gamma -W_{1}}{\alpha }}1_{0\leq \frac{y_{max}-W_{2}-W_{1}}{\alpha }}1_{H_{1}-H_{2}\leq y_{max}},\\ \; & I_{43}=\gamma (\min\left(x_{max},\frac{\gamma -W_{1}-W_{2}}{\alpha },\frac{y_{max}-W_{2}-W_{1}}{\alpha }\right)-\max\left(0,\frac{\left(\gamma -W_{1}\right)}{\alpha }\right)\\ \; & 1_{0\leq (\gamma -W_{1}-W_{2})}1_{0\leq \frac{y_{max}-W_{2}-W_{1}}{\alpha }}1_{x_{max}\geq \frac{\gamma -W_{1}}{\alpha }}1_{H_{1}-H_{2}\leq y_{max}},\\ \; & I_{44}=\alpha \frac{x^{2}}{2}+W_{1}x{|}_{lb_{44},ub_{44}}1_{0\leq (\gamma -W_{1}-W_{2})}1_{0\leq \frac{y_{max}-W_{2}-W_{1}}{\alpha }}1_{x_{max}\geq \frac{\gamma -W_{1}}{\alpha }}1_{H_{1}-H_{2}\leq y_{max}},\\ \; & ub_{42}=\min\left(x_{max},\frac{y_{max}-W_{2}-W_{1}}{\alpha },\frac{y_{max}-W_{1}}{\alpha }\right),\\ \; & lb_{42}=\max\left(0,\frac{y_{max}-W_{1}}{\alpha }\right),\\ \; & ub_{44}=\min\left(x_{max},\frac{y_{max}-W_{2}-W_{1}}{\alpha }\right),\\ \; & lb_{44}=\max\left(0,\frac{\gamma -W_{1}}{\alpha },\frac{\gamma -W_{2}-W_{1}}{\alpha }\right). \end{array}$$

## Appendix B

(A22) I2n+1,m,2=2P(S2n+1)(ΔXn)2tan(θcrn)∑i=14Ik,2n+1,2,i+(θnLm−1−θcrn)Λm(Lm−1).I2n+1,m,2,1=Gk,2n+1,2,1(θcrn)−Gk,2n+1,2,1(θnLm−1)G2n+1,m,1(θ)=cos(Θ)sin(β)akΔXnb(tan(θ)cos(2θ)+1+2σ2)2+1+σ2F(θ|11+σ2)−1+σ2E(θ|11+σ2)I2n+1,m,2=(amb2∑k=0∞12k+1−12k(Fsec2k+1(min(θ^,θcrn))−Fsec2k+1(θnLm−1)−amb2∑k=1∞−12k(Fcos2k(θcrn)−Fcos2k(min(θ^,θcrn)))12k+amb2ln(ΔXnb)(θcrn−min(θ^,θcrn))−(Flogcos(θcrn)−Flogcos(min(θ^,θcrn))))1ΔXnb<1(−amb2∑k=1∞−12k(Fcos2k(θcrn)−Fcos2k(θnLm−1))12k+amb2ln(ΔDnb)(θcrn−θnLm−1)−(Flogcos(θcrn)−Flogcos(θnLm−1)))1ΔXnb>1+amb2(θcrn−θnLm−1)ln(b).θ^=cos−1(ΔXnb)Fsec2k+1(x)=(tan(x)sec2k−1(x))∑n=0k(k−(2n−1)2)(n)(k−n)(n)cos2n(x)+ln(sin(x2)+cos(x2)cos(x2)−sin(x2))(−12)(k)(1)(k)Fcos2k(x)=(sin(x)cos2k−1(x))∑n=0k(k−(2n−1)2)(n)(k−n)(n)cos−2n(x)Flogcos(x)=−xln(2)+∑n=1∞(−1)n+1sin(2nx)2n2I2n+1,m,3=2P(S2n+1)(ΔXn)2tan(θcrn)asin(β)ΔXn(log(cos(θcrn2)+sin(θcrn2)cos(θcrn2)−sin(θcrn2))−log(cos(θnLm−12)+sin(θnLm−12)cos(θnLm−12)−sin(θnLm−12)),I2n+1,m,4=2P(S2n+1)(ΔXn)2tan(θcrn)bk2ΔXn(tan(θcrn)−tan(θnLm−1)).

with $a_{\left(n\right)}$ being the Pochhammer function.

(A23) I2n,m,2=2P(S2n+1)(ΔXn)2cot(θcrn)∑i=14Ik,2n+1,2,i+(θnLm−1−θcrn)Λm(Lm−1).I2n,m,2,1=Gk,2n+1,2,1(θcrn)−Gk,2n+1,2,1(θnLm−1)G2n,m,1(θ)=cos(Θ)sin(β)akΔXnb(tan(θ)cos(2θ)+1+2σ2)2+1+σ2F(θ|11+σ2)−1+σ2E(θ|11+σ2)I2n,m,2=(amb2∑k=0∞12k+1−12k(Fcosec2k+1(min(θ^,θcrn))−Fcosec2k+1(θnLm−1)−amb2∑k=1∞−12k(Fsin2k(θcrn)−Fsin2k(min(θ^,θcrn)))12k+amb2ln(ΔXnb)(θcrn−min(θ^,θcrn))−(Flogsin(θcrn)−Flogsin(min(θ^,θcrn))))1ΔXnb<1(−amb2∑k=1∞−12k(Fsin2k(θcrn)−Fsin2k(θnLm−1))12k+amb2ln(ΔDnb)(θcrn−θnLm−1))−(Flogsin(θcrn)−Flogsin(θnLm−1)))1ΔXnb>1+amb2(θcrn−θnLm−1)ln(b).θ^=cos−1(ΔXnb)Fsec2k+1(x)=(cot(x)cosec2k−1(x))∑n=0k(k−(2n−1)2)(n)(k−n)(n)sin2n(x)+ln(sin(x)cos(x))(−12)(k)(1)(k)Fsin2k(x)=(sin(x)cos2k−1(x))∑n=0k(k−(2n−1)2)(n)(k−n)(n)sin−2n(x)Flogsin(x)=−xln(2)−∑n=1∞(−1)n+1sin(2nx)2n2I2n+1,m,3=2P(S2n+1)(ΔXn)2cot(θcrn)asin(β)ΔXn(ln(sin(θnLm−1)cos(θnLm−1)))−ln(sin(θcrn)cos(θcrn))),I2n+1,m,4=2P(S2n+1)(ΔXn)2tan(θcrn)bk2ΔXn(cot(θcrn)−cot(θnLm−1)).

## Appendix C

(A24)
$$\begin{array}{cc} \; & A_{ip}^{1}=\Delta \theta \left(L_{p}^{2}-L_{p-1}^{2}\right)+\Delta \theta \ln\left(\left(L_{p}^{2}+d_{ip}^{2}\right)/\left(L_{p-1}^{2}+d_{ip}^{2}\right)\right)+\\ \; & 0.5\sum_{N=1}^{\infty }\left({\left(\frac{2d_{ip}L_{p}}{L_{p}^{2}+d_{ip}^{2}}\right)}^{n}-{\left(\frac{2d_{ip}L_{p-1}}{L_{p-1}^{2}+d_{ip}^{2}}\right)}^{n}\right){\left(-1\right)}^{n+1}(F_{\cos}^{n}\left(\left(m+1\right)\Delta \theta \right)-F_{\cos}^{n}\left(m\Delta \theta \right)\\ \; & -1_{\frac{\eta_{2}}{2}\frac{\sin((m+0.5)\Delta \theta )}{\eta_{1}\cos\left(\left(m+0.5\right)\Delta \theta \right)}<1}\eta_{2}(\Delta \theta +2\frac{\eta_{1}}{\sqrt{(}1-\eta_{1}^{2}}(\tanh^{-1}(\frac{\left(\eta_{1}-1\right)\tan\left(\left(m+1\right)\Delta \theta \right)}{\sqrt{1-\eta_{1}^{2}}}-\\ \; & \frac{\left(\eta_{1}-1)\tan(m\Delta \theta \right)}{\sqrt{1-\eta_{1}^{2}}})-1_{\frac{\eta_{2}}{2}\frac{\sin((m+0.5)\Delta \theta )}{\eta_{1}\cos\left(\left(m+0.5\right)\Delta \theta \right)}>1}\eta_{2}(\left(\frac{\pi }{2}-\frac{\eta_{1}}{\eta_{2}}\right)\ln\left(\frac{\left|\sin((m+1)\Delta \theta |\right)}{\left|\sin(m\Delta \theta )|\right)}\right)+\\ \; & \eta_{1}\left(\Delta \theta +\tan\left(\left(m+1\right)\Delta \theta \right)-\tan\left(m\Delta \theta \right)\right)\\ \; & \eta_{1}=\frac{L_{p}L_{p-1}+d_{ip}^{2}}{L_{p}^{2}+L_{p-1}^{2}}\\ \; & \eta_{2}=d_{ip}\frac{L_{p}-L_{p-1}}{L_{p}+L_{p-1}}\\ \; & A_{ip}^{2}=A_{ip}^{L_{p},\left(m+1\right)\Delta \theta }+A_{ip}^{L_{p-1},m\Delta \theta }-A_{ip}^{L_{p},m\Delta \theta }-A_{ip}^{L_{p-1},\left(m+1\right)\Delta \theta }\\ \; & A_{ip}^{L,\theta }=b(-d_{ip}\sin\left(\theta \right)\left(\tanh^{-1}\left(\frac{L+d_{ip}\cos\left(\theta \right)}{\sqrt{L^{2}+d_{ip}^{2}+2d_{ip}L\cos\left(\theta \right)}}\right)\right)\\ \; & -\left(L+d_{ip}\right)F\left(\frac{\theta }{2}|\frac{4d_{ip}L}{{\left(L+d_{ip}\right)}^{2}}\right)+\frac{\left(L^{2}+d_{ip}^{2}\right)}{L+d_{ip}}E\left(\frac{\theta }{2}|\frac{4d_{ip}L}{{\left(L+d_{ip}\right)}^{2}}\right)+\\ \; & 2\left(L+d_{ip}\right)F\left(\frac{\theta }{2}|\frac{4d_{ip}L}{{\left(L+d_{ip}\right)}^{2}}\right))+1_{|L+d_{ip}\cos\left(\theta \right)|<d_{ip}\sin\left(\theta \right)}\frac{\sin(\theta )}{\left|\sin(\theta )\right|}(\frac{-L^{2}}{2}d_{ip}\cos\left(\theta \right)+\\ \; & \frac{L^{2}}{8d_{ip}}\ln(|\tan\left(\frac{\theta }{2}\right)\left|\right)+\frac{L^{3}d_{ip}}{3}\ln(|\sin\left(\theta \right)\left|\right)+\frac{L^{2}d_{ip}}{4d_{ip}}(\cos\left(\theta \right)+\ln(|\tan\left(\frac{\theta }{2}\right)\left|\right)+\\ \; & 1_{|L+d_{ip}\cos\left(\theta \right)|>d_{ip}\sin\left(\theta \right)}\left(\frac{L^{3}}{3}\theta +\frac{L^{2}}{2}d_{ip}\sin\left(\theta \right)+\frac{Ld_{ip}^{2}}{4}\theta -\frac{Ld_{ip}^{2}}{8}\sin\left(2\theta \right)-\frac{d_{ip}^{3}}{2}R\left(L,\theta \right)\right)\\ \; & R\left(L,\theta \right)=\frac{\sin^{3}\left(\theta \right)}{3}\ln\left(d_{ip}\right)+\frac{\sin^{3}\left(\theta \right)}{3}\ln(|\frac{L}{d_{ip}}+\cos\left(\theta \right)\left|\right)+\\ \; & \frac{1}{3}\left(-\frac{\sin^{3}\left(\theta \right)}{3}-\frac{L}{d_{ip}}\frac{\sin(2\theta )}{4}+\left(\frac{L^{2}}{d_{ip}^{2}}-1\right)\sin\left(\theta \right)\right)\\ \; & +\frac{1}{3}\left(-\frac{L}{d_{ip}}\left(\frac{L^{2}}{d_{ip}^{2}}-1\right)\theta +\frac{L}{2d_{ip}}\theta \right)-\frac{2}{3}{\left(1-\frac{L^{2}}{d_{ip}^{2}}\right)}^{3/2}\tanh^{-1}\left(\frac{\left(\frac{L}{d_{ip}}-1\right)}{\sqrt{1\frac{L^{2}}{d_{ip}^{2}}}}\tan\left(\frac{\theta }{2}\right)\right)\\ \; & A_{ip}^{3}=A_{ip}^{L_{p},\left(m+1\right)\Delta \theta }+A_{ip}^{L_{p-1},m\Delta \theta }-A_{ip}^{L_{p},m\Delta \theta }-A_{ip}^{L_{p-1},\left(m+1\right)\Delta \theta }\\ \; & A_{ip}^{\theta ,L}=a(-d_{ip}\sin\left(\theta \right)\left(tanh^{-1}\left(\frac{L+d_{ip}\cos\left(\theta \right)}{\sqrt{L^{2}+d_{ip}^{2}+2d_{ip}L\cos\left(\theta \right)}}\right)\right)-\left(L+d\right)F\left(\frac{\theta }{2}|\frac{4d_{ip}L}{{\left(L+d_{ip}\right)}^{2}}\right)\\ \; & +\frac{\left(L^{2}+d_{ip}^{2}\right)}{L+d_{ip}}E\left(\frac{\theta }{2}|\frac{4dL}{{\left(L+d_{ip}\right)}^{2}}\right)+2\left(L+d_{ip}\right)F\left(\frac{\theta }{2}|\frac{4d_{ip}L}{{\left(L+d_{ip}\right)}^{2}}\right)) \end{array}$$

## Appendix D

(A25) $$\begin{array}{cc} \; & \alpha \in [-\cos^{-1}\left(u_{d_{i}}\left(L,\theta \right)\right)-\theta_{d_{i}}\left(L,\alpha \right),\cos^{-1}\left(u_{d_{i}}\left(L,\theta \right)\right)-\theta_{d_{i}}\left(L,\alpha \right)],\\ \; & 0\leq \theta_{l}^{+}\leq \pi ,L\leq B_{1,i,l}^{+}\\ \; & \alpha \in \left[-\pi ,\cos^{-1}\left(u_{d_{i}}\left(L,\theta \right)\right)-\theta_{d_{i}}\left(L,\alpha \right)\right]\cup \left[-\cos^{-1}\left(u_{d_{i}}\left(L,\theta \right)\right)-\theta_{d_{i}}{\left(L,\alpha \right)}_{2\pi },\pi \right],\\ \; & 0\leq \theta_{l}^{+}\leq \pi ,B_{1,i,l}^{+}\leq L\leq B_{2,i,l}^{+}\\ \; & \alpha \in [-\cos^{-1}\left(u_{d_{i}}\left(L,\theta \right)\right)-\theta_{d_{i}}\left(L,\alpha \right),\cos^{-1}\left(u_{d_{i}}\left(L,\theta \right)\right)-\theta_{d_{i}}\left(L,\alpha \right)],\\ \; & 0\leq \theta_{l}^{+}\leq \pi ,B_{2,i,l}^{+}\leq L\\ \; & \alpha \in [\cos^{-1}\left(u_{d_{i}}\left(L,\theta \right)\right)-\theta_{d_{i}}{\left(L,\alpha \right)}_{2\pi },-\cos^{-1}\left(u_{d_{i}}\left(L,\theta \right)\right)-\theta_{d_{i}}{\left(L,\alpha \right)}_{2\pi }],\\ \; & \pi \leq \theta_{l}^{+}\leq \frac{3\pi }{2},L\leq B_{1,i,l}^{+}\\ \; & \alpha \in \left[-\pi ,\cos^{-1}\left(u_{d_{i}}\left(L,\theta \right)\right)-\theta_{d_{i}}\left(L,\alpha \right)\right]\cup \left[-\cos^{-1}\left(u_{d_{i}}\left(L,\theta \right)\right)-\theta_{d_{i}}{\left(L,\alpha \right)}_{2\pi },\pi \right],\\ \; & \pi \leq \theta_{l}^{+}\leq \frac{3\pi }{2},B_{1,i,l}^{+}\leq L\leq B_{2,i,l}^{+}\\ \; & \alpha \in [-\cos^{-1}\left(u_{d_{i}}\left(L,\theta \right)\right)-\theta_{d_{i}}{\left(L,\alpha \right)}_{2\pi },\cos^{-1}\left(u_{d_{i}}\left(L,\theta \right)\right)-\theta_{d_{i}}{\left(L,\alpha \right)}_{2\pi }],\\ \; & \pi \leq \theta_{l}^{+}\leq \frac{3\pi }{2},B_{2,i,l}^{+}\leq L\\ \; & \alpha \in [-\cos^{-1}\left(u_{d_{i}}\left(L,\theta \right)\right)-\theta_{d_{i}}\left(L,\alpha \right),\cos^{-1}\left(u_{d_{i}}\left(L,\theta \right)\right)-\theta_{d_{i}}\left(L,\alpha \right)],\\ \; & \theta_{l}^{+}\geq \frac{3\pi }{2},L\leq B_{1,i,l}^{+}\\ \; & \alpha \in \left[-\pi ,\cos^{-1}\left(u_{d_{i}}\left(L,\theta \right)\right)-\theta_{d_{i}}{\left(L,\alpha \right)}_{-2\pi }\right]\cup \left[-\cos^{-1}\left(u_{d_{i}}\left(L,\theta \right)\right)-\theta_{d_{i}}\left(L,\alpha \right),\pi \right],\\ \; & \theta_{l}^{+}\geq \frac{3\pi }{2},B_{1,i,l}^{+}\leq L\leq B_{2,i,l}^{+}\\ \; & \alpha \in [-\cos^{-1}\left(u_{d_{i}}\left(L,\theta \right)\right)-\theta_{d_{i}}\left(L,\alpha \right),\cos^{-1}\left(u_{d_{i}}\left(L,\theta \right)\right)-\theta_{d_{i}}\left(L,\alpha \right)]\\ \; & \theta_{l}^{+}\geq \frac{3\pi }{2},B_{2,i,l}^{+}\leq L \end{array}$$

while the solution for the case under patching is

(A26) $$\begin{array}{cc} \; & \alpha \in [-\cos^{-1}\left(u_{d_{i}}\left(L,\theta \right)\right)-\theta_{d_{i}}\left(L,\alpha \right),\cos^{-1}\left(u_{d_{i}}\left(L,\theta \right)\right)-\theta_{d_{i}}\left(L,\alpha \right)],\\ \; & 0\leq \theta_{l}^{+}\leq \pi ,L\leq B_{1,i,l}^{-}\\ \; & \alpha \in \left[-\pi ,\cos^{-1}\left(u_{d_{i}}\left(L,\theta \right)\right)-\theta_{d_{i}}\left(L,\alpha \right)\right]\cup \left[-\cos^{-1}\left(u_{d_{i}}\left(L,\theta \right)\right)-\theta_{d_{i}}{\left(L,\alpha \right)}_{2\pi },\pi \right],\\ \; & 0\leq \theta_{l}^{+}\leq \pi ,B_{1,i,l}^{-}\leq L\leq B_{2,i,l}^{-}\\ \; & \alpha \in [-\cos^{-1}\left(u_{d_{i}}\left(L,\theta \right)\right)-\theta_{d_{i}}\left(L,\alpha \right),\cos^{-1}\left(u_{d_{i}}\left(L,\theta \right)\right)-\theta_{d_{i}}\left(L,\alpha \right)],\\ \; & 0\leq \theta_{l}^{+}\leq \pi ,B_{2,i,l}^{-}\leq L\\ \; & \alpha \in [-\cos^{-1}\left(u_{d_{i}}\left(L,\theta \right)\right)-\theta_{d_{i}}\left(L,\alpha \right),\cos^{-1}\left(u_{d_{i}}\left(L,\theta \right)\right)-\theta_{d_{i}}\left(L,\alpha \right)],\\ \; & \theta_{l}^{+}\geq \pi ,L\leq B_{1,i,l}^{-}\\ \; & \alpha \in \left[-\pi ,\cos^{-1}\left(u_{d_{i}}\left(L,\theta \right)\right)-\theta_{d_{i}}{\left(L,\alpha \right)}_{-2\pi }\right]\cup \left[-\cos^{-1}\left(u_{d_{i}}\left(L,\theta \right)\right)-\theta_{d_{i}}\left(L,\alpha \right),\pi \right],\\ \; & \theta_{l}^{+}\geq \pi ,B_{1,i,l}^{-}\leq L\leq B_{2,i,l}^{-}\\ \; & \alpha \in [-\cos^{-1}\left(u_{d_{i}}\left(L,\theta \right)\right)-\theta_{d_{i}}\left(L,\alpha \right),\cos^{-1}\left(u_{d_{i}}\left(L,\theta \right)\right)-\theta_{d_{i}}\left(L,\alpha \right)],\\ \; & \theta_{l}^{+}\geq \pi ,B_{2,i,l}^{-}\leq L \end{array}$$
